# Transformer-Driven Explainable Deep Learning with Quantitative Attribution Validation for Liver Tumor Detection

**DOI:** 10.3390/bioengineering13060616

**Published:** 2026-05-25

**Authors:** Inzamam Mashood Nasir, Hend Alshaya, Sara Tehsin, Wided Bouchelligua

**Affiliations:** 1 Human-Environment-Technology (HET) Systems Centre, Mykolas Romeris University, 08303 Vilnius, Lithuania; 2 Applied College, Imam Mohammad Ibn Saud Islamic University (IMSIU), Riyadh 11432, Saudi Arabia; hialshaya@imamu.edu.sa (H.A.); wabouchelligua@imamu.edu.sa (W.B.); 3 Faculty of Informatics, Kaunas University of Technology, 51368 Kaunas, Lithuania; sara.tehsin@ktu.edu

**Keywords:** liver tumor detection, explainable AI, transformer models, attribution mapping, CT imaging

## Abstract

The identification of liver tumors on computed tomography (CT) scans is hindered by myriad factors, including tumor heterogeneity, anatomical variability, and the limited interpretability of deep learning models in clinical settings. The present research introduces a deep learning-based framework, referred to as the ‘form of the Transformer’, in combination with Global Context (GC) fused with Transformer (Tf) and the Quantitative Attribution (QA) module, for a first reliable, explainable liver tumor detection framework. Moving away from traditional opaque classification systems, this framework uses gradient-based attribution with a localization module and evaluates its spatial alignment with tumor annotations without requiring segmentation supervision during model training. The framework accounts for long-range spacing and leverages Tf-Encoders, which substantially improve the system’s tumor-detection performance. Integrating the Attribution, this framework significantly enhances Qualitative Evidence (QE) in clinical settings. The experimental study has shown strong classification performance with the following metrics: accuracy 96.9%, precision 96.2%, recall 95.8%, F1-score 96.0%, area under the receiver operating characteristic curve 97.6%, and Matthews correlation coefficient 0.93. The classification-based localization of the system achieves an Intersection over Union (IoU) of 71.6% and a Dice coefficient of 83.5%, underscoring the alignment of tumor regions with their attributions. The results indicated significant improvements over existing CNN- and TF-based systems.

## 1. Introduction

Liver cancer is a major global health concern, and recent studies discuss the potential of artificial intelligence (AI) to transform the study, identification, and treatment of liver cancer, integrated into the workflows of radiology and oncology [1]. AI-based imaging tools are already helping streamline and individualize liver disease evaluations by minimizing variability introduced by the observer and identifying imaging biomarkers otherwise undetectable by the human observer [2]. Meanwhile, recent surveys in liver imaging have reported that modern AI systems are being applied to lesion detection, tumor characterization and segmentation, radiomics, treatment planning, and prognosis, demonstrating the wide scope of this field and a significant demand for relevant technologies [3]. In liver cancer care, a large scoping review reported that the most effective AI technology is deep learning and underscored continued challenges, particularly in externally validating, defining specific tasks, and addressing translational applicability [4,5,6].

In methodological terms, early post-2023 liver classification studies continued to show that transfer learning, especially the reuse of pre-trained convolutional encoders, can yield good diagnostic results, even with reduced datasets, for CT-based tumor classification [7]. However, transfer learning pipelines for small cohorts tend to be biased toward natural imaging features and thus may not sufficiently address the full heterogeneity of liver tumors, the various enhancement patterns, and the surrounding parenchyma. The classification effort focused on feature engineering remained pertinent, and recent studies have attempted to combine handcrafted features with deep neural networks for CT imaging of liver tumors. Despite the potential for such hybrid models to enhance classification within the scope of curated datasets, they remain reliant on pre-processing heuristics and manually crafted features, which negatively impact reliability when acquisition settings and lesion appearances vary across patients and scanners [8].

Certain clinically oriented diagnostic frameworks that focus on re-creating real-world scenarios have gone beyond the need for fixed input assumptions by utilizing multiphase CT imaging and the incomplete availability of imaging for liver analyses [9,10]. Using this approach, a flexible deep learning framework has shown a solid AUROC for distinguishing between hepatocellular carcinoma, intrahepatic cholangiocarcinoma, and healthy patients while also accommodating missing phases in the analysis. Another related multicentric study presented and validated a deep learning multistage CT system for the diagnosis of focal liver lesions from a large multicentric database and illustrated that the field is advancing toward large-scale multicentric evidence beyond a few single-centered small-scale studies [11,12]. Nevertheless, the advanced models remain modality-specific and imager-specific, and depend on highly curated imaging streams, making them difficult to replicate and less straightforward to adapt to publicly available slice-level datasets.

Recent studies on liver tumor baseline analysis have begun to explore frameworks that intertwine the processes of detection, delineation, and classification. For instance, a study [13] on an automated liver tumor detection and delineation tool via deep learning on CT scans of cancer patients demonstrated high patient-wise detection accuracy and strong performance at the lesion level, highlighting the possibility of highly automated, large-scale analysis of liver tumors. However, segmentation-based systems depend on precise lesion annotations and focus more on delineation than on providing diagnostic reasoning at the image level, unlike classification-based clinical decision support systems, which aim to classify images and support clinical decisions. As for classification, the newly proposed UNet70 framework [14,15] was trained on CT scans of patients with and without tuberculosis infection and achieved reasonable accuracy, demonstrating the potential of U-Net-based encoders for liver tumor classification. Yet, these classification models largely embody the key issue that most deep learning models deployed in healthcare are known for: the lack of explanation, especially when it is unclear whether the determination is based on the lesion or on the surrounding anatomical context.

The issue of interpretability has sparked new interest in explainable AI when applied to liver imaging. One recently proposed explainable deep learning model for the classification of multiparametric MRIs of focal liver lesions shows that explainability can be integrated into lesion classification rather than included as an afterthought [16]. Similarly, in the multi-sequence MRI study for the classification of focal liver lesions, attention-guided fusion and augmentation techniques were proposed to help consolidate the cross-sequence data, and the study further emphasized the importance of learning spatially meaningful liver lesions [17]. Moreover, explainability in liver imaging segmentation has shown that visual explanation maps can be used to understand the model’s different sensitivities; however, it has also been shown that qualitative heatmaps that are not part of a rigorous, anatomically relevant evaluative framework fall short on their own [18]. More generally, in the recent literature on explainable AI applied to radiomics, it has been noted that the field has yet to make substantive progress in establishing criteria used to evaluate explanatory faithfulness, stability, and clinical utility, particularly when post hoc saliency mapping is used to support clinically consequential diagnostic assertions [19].

The last few years have seen significant changes in medical imaging research, particularly with large-scale pre-trained representations and foundational models. A 2024 perspective on cancer imaging biomarkers noted that foundational models could diminish reliance on extensive task-specific annotations while supporting oncological imaging analysis via adjustable representations [20,21]. Further discussions on quantitative tumor imaging biomarkers for cancer research suggested that, when choosing a pretrained backbone for cancer imaging, particularly for robust and salient model behavior, downstream predictive performance should also be considered [22]. At a survey level, emerging studies have asserted that, for medical foundation models, trust-related concerns such as robustness, privacy, fairness, and explainability should be prioritized over other, more peripheral issues for safe and effective clinical application [23]. Similar conclusions have been reached in radiology-focused reviews, suggesting that while the outputs of foundational models and generative AI have the potential to improve imaging workflows, this will only be possible when appropriate clinical context, auditing, and interpretation are applied to the workflows [24].

Recent advancements in liver cancer analysis suggest a need to shift from predictive classification tools toward the integration of interpretable reasoning, personalized diagnostics, and image-based clinical validation. In medical image processing, recent advancements in deep learning, including convolution-based and detection-centric transformer approaches, have been shown to improve liver and hepatocellular carcinoma imaging by accurately localizing lesions, optimizing anatomical imaging, and discriminating tumor regions. Nonetheless, these models must include stronger interpretative components to clarify whether their reasoning is toward tumor evidence or contextual anatomical evidence. Simultaneously, current liver biomarker studies are focused on developing decentralized, quantified diagnostic technologies that leverage advanced imaging and the estimation of clinically relevant liver biomarkers, enabling early interventions for the management of hepatic disorders [25,26]. These findings are consistent with the integration of artificial intelligence technologies in health diagnostics and therapeutics, where AI-based diagnostics must provide not only accurate predictions but also transparent, logically grounded, and clinically verifiable rationales. Thus, explainable AI is critical in liver cancer imaging, as it helps interpret model reasoning, validate clinically relevant findings, and build trust in computer-aided diagnosis. Within this context, the current model offers a novel approach by combining transformer-based feature extraction with validated quantitative attribution, thereby establishing a link between classification and the reliability of spatial explanations, rather than relying solely on qualitative heatmap visualization.

The primary contributions of this study can be delineated as follows. We present a novel explainable deep learning framework for liver tumor detection that conceptualizes the task as a classification problem and enables interpretability while bypassing the need for bounding-box supervision via segmentation. Second, we propose a transformer-centric feature-encoding approach that captures global relational features and yields greater discriminative power than traditional convolution-based architectures. Third, we propose a quantitative interpretability evaluation framework for XAI that systematically quantifies the similarity between attribution-based localization and ground truth, facilitating the evaluation of the explainable rationales of machine learning algorithms. Fourth, we have established a solid baseline for all competing classification and localization algorithms implemented on the provided datasets, as the proposed method has been shown to be consistent, reproducible, and superior. Additionally, we provide comprehensive compositional and descriptive analyses of the XAI for each framework component to demonstrate how each component enhances the proposed approach and substantiates its value and consistency.

The rest of the paper is structured as follows. Section 2 discusses previous research, and Section 3 explains our methodology. Section 4 details the empirical evidence and associated commentary, while Section 5 concludes the paper.

## 2. Literature Review

Deep learning methods, particularly through convolutional neural networks (CNNs), have greatly improved the detection of liver cancer in computed tomography (CT) imaging. The initial approaches centered on patch-based CNNs for tumor classification and performed well at detecting liver lesions in CT slices. Ref. [27] presented a CNN framework for automatic liver tumor detection and reported good results in terms of classification accuracy by learning sufficient spatial features from the medical images. While advancements have been made in this area, the limitations of these approaches stem from their reliance on local receptive fields and a lack of global context, leading to poor generalization. To overcome the challenges stated, several researchers have considered using deeper CNNs and transfer learning. In [28], a liver tumor classification model using the ResNet framework was able to achieve better representation of features and improved classification using residual learning. The classification accuracy [29] also improved, as highlighted in the classification tasks of models using DenseNet, thanks to better feature reuse and gradient flow. Still, all techniques relying on CNNs face the same inherent limitation in modeling long-range relationships, which are crucial for capturing tumor structures and the varying liver anatomy.

More recently, the introduction of attention mechanisms has improved feature learning across various medical imaging tasks. An attention-based CNN model focuses on salient regions of tumors and achieves 95.5% accuracy in liver tumor classification [30]. Although attention modules can enhance feature localization, they are typically used within CNN backbones, resulting in models that lack the capacity to effectively capture global interactions. Attention-based models often lack attention maps that are not readily available for clinical review against ground truth, further limiting the model’s utility. More recently, transformer-based models have proven to be a viable and powerful alternative to the existing methods of analyzing medical images. The self-attention mechanisms in the Vision Transformer (ViT) architecture, which model features at a global level, effectively capture complex spatial relationships in images [31]. In this regard, Swin Transformer models have been used to analyze liver tumors and, by using hierarchical feature representations, achieved classification accuracy in the 94–96% range [32]. Despite the promising results, transformer-based models have disadvantages, such as the need for large-scale, resource-intensive training datasets, the lack of self-interpretation mechanisms, and the pressing need for models that can be interpreted in a medical setting.

Models that employ hybrid approaches integrating CNNs and transformers can approximate and reason locally and globally about attributes. An architecture based on UNet70 merges the Convolutional Encoder and Transformer-with-Most components with sufficient disparity to provide high classification performance [14]. Despite hybrid approaches exhibiting greater accuracy, they entail greater architectural complexity and additional computational burden, potentially hindering their economic feasibility for implementation in genuine clinical systems. Also, the analysis of liver tumors using segmentation-based methods, particularly with datasets from the Liver Tumor Segmentation Challenge (LiTS), has been thoroughly investigated. A multi-scale CNN framework demonstrated the efficacy of hierarchical feature extraction in segmentation tasks with a tumor Dice score of 72.5% and a liver Dice score of 96.7% [33]. Unfortunately, in segmentation models, achieving high segmentation performance requires abundant pixel-level annotations, which are both costly and labor-intensive to produce. Furthermore, segmentation performance is not necessarily predictive of classification performance, especially when only image-level annotations are available. The classification of liver tumors using lightweight CNN-based models has emerged recently. LiverCompactNet is a model that combines a compact CNN and achieves high classification performance while converging to lower computational cost [34]. As a result of the aforementioned models, an increase in efficacy is attained; however, they often rely on a multitude of parameters, and perhaps, most alarmingly, they lose any robust interpretability frameworks and are ultimately prized for their classification performance, without regard for the distributional proximity of the model’s predictions to the tumor regions.

The incorporation of XAI methods to enhance the understanding of deep learning models in medical imaging is a growing trend. An example of an XAI technique is Grad-CAM [35]. This technique combines several gradient-based methods to produce a ‘heatmap’ that shows the areas of a medical image that contribute to a model’s decision. Reference [36] documents the various medical image analysis tasks that Grad-CAM has provided visual explanations to in support of medical image classification. However, much of the literature has relied on qualitative visualizations and a dwindling number of medical imaging tasks as the basis for their explanations. This results in a lack of a clinical basis for their use. The literature has only recently begun to stress the need for quantitative evaluations of XAI methods, and attribution maps have begun to serve this purpose [37]. XAI is rarely integrated into the training of models with explainable components, typically being added as a secondary feature, but most of the literature investigates XAI as a primary feature of a model. Recent baseline methods, their performance, and their limitations are shown in Table 1.

Deep learning-based liver tumor analysis, including classification and segmentation, has advanced significantly in recent years, particularly with advances in convolutional and transformer architectures. However, several issues remain unresolved. Existing works focus on enhancing segmentation/classification tasks, while ignoring important aspects such as interpretability and trustworthiness in the clinical realm, creating a void in applicability. CNN approaches have too many limitations in receptive fields and in their ability to capture tumor complexity, due to their reliance on contextual associations. While transformers improve on these, most are black-box approaches evaluated solely on predictive performance, and when attention is considered, there are no methods to determine whether it is on clinically significant aspects. Heatmap-based explainability is the most common approach, but it lacks formal evaluation against ground truth. Finally, most works base their approaches on segmentation and rely on highly expensive pixel-level annotations, a void in clinical settings. All of the previously stated issues demand a more unified approach that combines high classification levels with the trustworthiness of well-supported interpretability.

The proposed approach seeks to address these problems by implementing a classification-oriented, explainable framework. Using a transformer-based encoder, the proposed model addresses the concerns about long-distance relationships that previous CNN models raised. The proposed formulation circumvents reliance on segmentation masks by treating tumor detection as a classification problem, while still allowing spatial localization via gradient-based attribution. A major aspect of this work is the first quantitative assessment of attribution, in which the masks produced by attribution are compared to ground-truth masks using overlap-based metrics. This ensures that the framework’s interpretability is not only qualitatively and statistically defensible but also reliable. In this work, for the first time, the attribution localizing maps, combined with consistency evaluation, help clarify the disparity between prediction and explanation, enabling the first complete estimation of the model. With this approach, the proposed model not only increases predictive power but also enhances the explainable AI’s confidence in the predictive model, thereby fulfilling all the objectives the proposed framework was intended to address.

## 3. Proposed Methodology

**Figure 1:**
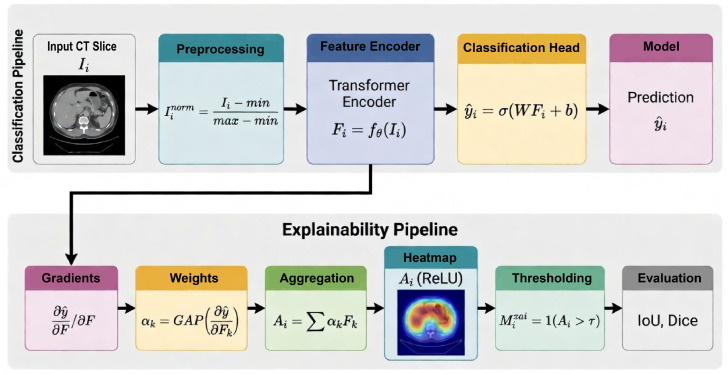
Overall framework of the proposed explainable liver tumor detection model.

The proposed framework for liver tumor detection presents an innovative approach by combining state-of-the-art explainable artificial intelligence (XAI)-based liver tumor classification systems with retraining systems that leverage model-agnostic attribution methods, providing insights into how input features contribute to a model’s predictions. The system incorporates transformer-based feature encoding—where transformers are neural network architectures designed to capture global contextual dependencies—and a model attribution framework. The framework operates on preprocessed computed tomography (CT) image slices, medical images obtained from CT scans, and reformulates tumor detection as a binary classification problem using an exhaustive attribution framework. Each component is tailored to maintain consistency between the system’s predictions and its explanations (predictive-explanatory consistency), supporting both high diagnostic accuracy and reliable XAI. Overall, the framework combines advanced liver tumor classification with robust XAI, addressing clinically relevant limitations in assessing how models behave in tumor-detection scenarios. The process flow for the proposed framework is shown in Figure 1, illustrating the integration of the classification and attribution pipeline to maintain predictive accuracy and verifiable XAI.

### 3.1. Problem Formulation

This task is approached as a supervised learning challenge, with the objective of enabling a model to learn to detect liver tumors in two-dimensional computed tomography slices while also supporting spatial interpretability via attribution methods. Let the dataset consist of image triplets, pixel-wise annotations, and image-wise labels. The formulation clarifies the distinction between classification objectives and local evaluation of spatial interpretability, ensuring the learning process remains prediction-focused while interpretability is assessed externally. It is assumed that each individual sample is drawn from a distribution of the underlying medical imaging data, where diversity arises from anatomical variability, imaging acquisition protocols, and tumor diversity. The goal is to learn a mapping that is applicable across all the aforementioned variations and sensitive to clinically significant areas. The formulation integrates both probabilistic prediction and spatial attribution and is the first to connect classification and interpretability. The assumptions underlying modeling are that tumor presence creates distinctive and discriminative imaging patterns that can be learned from deep features and interpreted using gradient-based attribution methods. Therefore, the problem is defined as a joint space of prediction accuracy and spatial localizability, where both features are vital for the model’s overall reliability.

(1) $$D={\left\{\left(I_{i},M_{i},y_{i}\right)\right\}}_{i=1}^{N}$$

Here, *N* is the total number of samples. $I_{i}\in R^{H\times W}$ indicates the input CT (computed tomography) slice, where *H* and *W* are the height and width of the image. $M_{i}\in {\left\{0,1\right\}}^{H\times W}$ denotes the binary tumor mask, a matrix indicating the presence (1) or absence (0) of tumor pixels in each position. $y_{i}\in \left\{0,1\right\}$ is a binary label that represents tumor presence in the slice, meaning $y_{i}=1$ if at least one pixel in $M_{i}$ is 1, and $y_{i}=0$ if all pixels in $M_{i}$ are 0. The dataset is considered independent and identically distributed (iid), though variability between scanners and patients can cause domain shifts, that is, changes in the underlying data distribution. In this context, $M_{i}$ is not used to train the classifier but rather to evaluate how well attribution maps, which highlight regions influencing the model’s prediction, align spatially with tumor areas. By not using $M_{i}$ during training, the approach avoids relying on pixel-level supervision and maintains focus on the overall classification task. This dataset structure supports clear definitions of both predictive objectives (detecting tumor presence) and interpretability objectives (assessing attribution spatial consistency), thereby integrating both goals into the process of model learning and evaluation.

(2) $$y_{i}=⊮\left(\sum_{u=1}^{H}\sum_{v=1}^{W}M_{i}\left(u,v\right)>0\right)$$

A mapping function refers to a mathematical method that assigns each input to an output, defining how parameterized models interpret and process input images to compute probability values. Here, θ is the model’s adjustable parameter, and $f_{\theta }$ is the resulting function that produces a single number between 0 and 1, expressing the estimated probability of a tumor being present in the image. The objective function is a specific formula that performs a nonlinear transformation, ensuring the model’s output always remains a valid probability. The model is assumed to effectively capture detailed visual patterns in small regions (local texture) and in larger regions (global structure) of the image, both of which are necessary for reliable tumor detection. As the output represents a probability, widely used loss functions (mathematical measures of prediction error) are applicable, and the model’s accuracy can be evaluated using standard thresholds that decide when to classify an image as tumor-positive or -negative. The mapping function’s differentiability means it can be optimized using algorithms that compute gradients—allowing the model to learn from data—and also supports attribution analytics, which help explain the contribution of different input features. Thus, the relationship between input and output is understood as a two-step process: extracting relevant features from the image and making a decision, which together define a classification model.

(3) $${\hat{y}}_{i}=f_{\theta }\left(I_{i}\right)$$

The goal of this optimization process is to minimize the difference between predicted probabilities and actual labels. A binary cross-entropy formulation is selected because it is well-suited to classify the problem as a probabilistic one. The loss function is designed to promote correct classifications and to punish incorrect classifications with low confidence. The expectation is that the learned parameters will generalize to the entire dataset and not to individual samples. The formulation also assumes that the dataset is large enough to capture the variability of the appearances of liver tumors. The optimization is performed using gradient-based methods, and the differentiability of the model and loss functions is key. The loss function stabilizes the model’s learning by guiding it toward a correct state. The resulting goal function serves as a proper baseline for estimating the parameters of a binary-class model.

(4) $$L_{cls}=-\frac{1}{N}\sum_{i=1}^{N}\left[y_{i}\log\left({\hat{y}}_{i}\right)+\left(1-y_{i}\right)\log\left(1-{\hat{y}}_{i}\right)\right]$$

The proposed formulation involves both prediction and interpretability via attribution mapping. Let $A_{i}\in R^{H\times W}$ be the attribution map associated with the input image $I_{i}$ and captures how each pixel contributes to the decision of the model. Attribution maps are computed as the gradients of the prediction with respect to the model’s layers, showing how perturbed outputs are with respect to the input. The formulation posits that for larger values of $A_{i}$, the corresponding regions are more important in determining the class to which the input image belongs. The attribution mechanism aligns with the model’s architecture, providing explanations that remain faithful to its representations. The synthesis of attribution in the formulation enables the assessment of predictive accuracy and its spatial relevance. This is especially important in biomedical imaging, where interpretability is paramount.

(5) $$A_{i}=\frac{\partial {\hat{y}}_{i}}{\partial I_{i}}$$

To compute the model’s interpretability, the attribution map, which highlights the contribution of each input element to the model’s prediction, is thresholded to produce a binary localization mask. Let τ be the threshold that separates the signal of interest (i.e., areas the model considers important) from background noise. The localization mask captures the model’s focus—or the regions it attends to—and is evaluated by measuring its overlap with the ground truth, which represents the desired or accurate outcome. Thresholding helps assess sensitivity (the ability to detect true signals) and specificity (the ability to ignore noise), but involves balancing both metrics. The mask is formulated to remain consistent with the attribution map while providing a binary mask ready for evaluation. The map-to-mask process translates the continuous range of attribution values into discrete anatomical structures for assessment.

(6) $$M_{i}^{xai}=⊮\left(A_{i}>\tau \right)$$

The last element of the formulation considers the alignment of the mask obtained from attribution with the ground truth tumor annotations. Alignment is assessed using overlap metrics that quantify the spatial correspondence between the predicted and actual regions. The evaluation uses the Intersection over Union metric, which is the most robust for assessing the overlap of binary masks. The formulation captures the model’s attention to clinically pertinent areas, quantifying its interpretability. The evaluation is done separately from the training goal so that interpretability evaluations do not influence the learning process. The metric obtained provides a meaningful assessment of the model’s alignment between internal reasoning and medical annotations, thereby enhancing the model’s trustworthiness.

(7) $${\mathrm{IoU}}_{i}=\frac{|M_{i}^{xai}\cap M_{i}|}{|M_{i}^{xai}\cup M_{i}|}$$

Because the attribution map is based on these gradients (which indicate how each input value affects the model’s output), it can be inferred that areas with higher attribution values correspond to tumor locations. The thresholding operation preserves these high-attribution areas while suppressing regions of low relevance, producing a binary mask (a two-value map) that captures the tumor annotation. Thus, within these parameters, the localization mask aligns with the ground truth (actual tumor locations as labeled in the data).

The proposed framework purposely redefines the analysis of liver tumors as a classification problem, rather than a fully supervised segmentation problem. This is primarily due to challenges in obtaining detailed medical annotations. Obtaining detailed medical annotations is expensive, time-consuming, and not always available in real-world medical practices. The framework combines attribution-based localization consistency analysis, which provides spatial interpretability without segmentation supervision. This harnesses the framework’s annotation dependency and solves the problem of indicator dependence. It also allows for the assessment of regional focus Lewy stressors in a clinically relevant case. The framework is therefore a clinically viable, computationally efficient, and practically useful compromise between the three components: predictive power, explainability, and annotation scope.

### 3.2. Dataset and Preprocessing Pipeline

**Figure 2:**
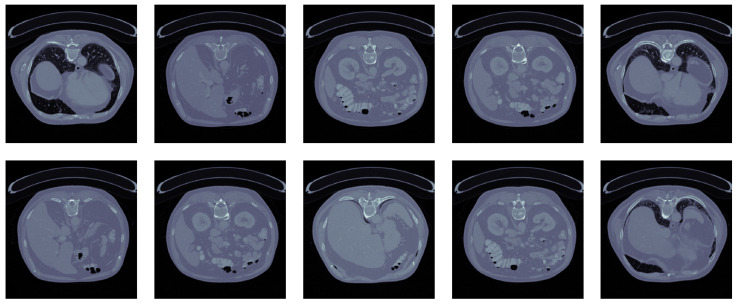
Representative samples from the CT-based liver tumor dataset used in this study.

The Liver Tumor Segmentation Benchmark (LiTS) and 3DIRCADb datasets were used for the experiments. These datasets contain annotated CT scans of the liver. CT scans were split into smaller scans called slices. Each slice was labeled tumor-positive or tumor-negative based on the presence of a tumor. Tumor masks were used to evaluate the localization of tumor scans. Attribution maps refer to the localization maps generated by the system. The goal of the system is to provide an explanation of the reasoning behind a diagnosis. The system was designed not to rely on tumor quality or detailed tumor masks during training or evaluation. The preprocessing pipeline included intensity normalization, spatial resizing, filtering scans containing irrelevant anatomical structures, and CT dataset standardization to compensate for differences in acquisition protocols and scanner equipment. Each CT slice was individually normalized using a min–max approach and then resized. CT slices were standardized using dataset-level statistical parameters. Slices containing irrelevant anatomical structures of the liver were removed. This was done to improve the signal-to-noise ratio and stabilize the training process. Examples of CT slices from the dataset, showing the variability in tumor appearance and surrounding anatomy, are presented in Figure 2.

The initial stage of the process provides a consistent representation space for the subsequent model to learn discriminative features. Furthermore, the pipeline removes excess non-informative samples through spatial standardization and region filtering. The formulation means that the preprocessing transforms the raw input into a normalized domain while preserving the original anatomical information. Thus, the resulting dataset representation is optimal for both classification and consistent attribution. The entire process is deterministic, ensuring stability and reproducibility across the entire training series. This step is critical for minimizing the gap between the input data distribution and the learning model’s assumptions.

(8) $$D^{prep}={\left\{\left(I_{i}^{prep},M_{i},y_{i}\right)\right\}}_{i=1}^{N^{\prime }}$$

Each CT slice is first normalized to a fixed intensity range using min–max normalization, reducing the impact of varying intensity scales across scans for more reliable optimization. Slice normalization is performed individually prior to standardizing the entire image. Each slice’s minimum and maximum intensity values are calculated, enabling adaptive scaling to local variations in tissue density. For instance, extremely high voxel intensities in a CT slice are encountered in bone, and air is preserved in the resulting image, whereas other high values are limited. In clinical imaging, this is of utmost importance to avoid overemphasizing certain anatomical regions, thereby losing low-intensity structures. Many volumetric CT imaging/diagnosing processes rely on transformer networks, where maintaining input values within a specific range is crucial for the underlying neural network layers to function optimally. The resulting images contain the inherent variability of the original images and are of extreme importance for increasing the network’s convergence rate and improving the efficiency of subsequent clinical imaging processing stages and downstream computations.

(9) $$I_{i}^{norm}=\frac{I_{i}-\min\left(I_{i}\right)}{\max\left(I_{i}\right)-\min\left(I_{i}\right)}$$

After normalization, all slices are adjusted to a consistent spatial size of H×W. This is done to ensure that all inputs in the dataset have the same dimensions. Changing the image dimensions is achieved using interpolation methods that preserve the image’s spatial coherence. We define the resizing operation R(·) as resampling the normalized image onto a spatially fixed grid. This is a required step because deep learning models operate on fixed dimensions to enable batch-size splitting and weight sharing. In addition to improving memory utilization, fixed dimensions simplify computational processes during model training. Keeping the same resizing strategy across the entire dataset ensures that anatomical structures, including tumors, maintain spatial coherence and are uniformly distorted. Optimizing the computational dimensions to enable the model to operate without unnecessary overhead captures the pertinent features. The initial representation of the image the model uses throughout the learning process is obtained by resizing the image to the chosen fixed dimensions. In addition to helping the model learn image features, resetting the image dimensions spatially normalizes all data samples to a unified coordinate system, ensuring the model’s feature extraction process is consistent.

(10) $$I_{i}^{res}=R\left(I_{i}^{norm}\right)$$

To further streamline the dataset, we exclude slices that do not contain any defined liver anatomical regions from the training set. This not only reduces the irrelevant background data in the dataset, but also improves its signal-to-noise ratio. Let $\phi (I_{i}^{res})$ be a function that determines if a slice contains a sufficient amount of liver tissue in it. Using the established filtering criteria, only the diagnostically relevant slices are retained, aiming to reinforce learning of more significant patterns. This is a critical step in training the model, since several slices from the volumetric CT scans are likely from regions outside the liver and do not aid tumor detection. This also reduces the risk of the model learning irrelevant or trivial patterns and steers it towards the relevant parts of the anatomy. The approximation of the dataset, $N^{\prime }$, always satisfies the constraint $N^{\prime }\leq N$. This decreases the dataset size, and with the refined dataset, the training and model generalization are enhanced. The filtering system is deterministic and hence reproducible, ensuring it produces the same results across multiple tests.

(11) $$I_{i}^{filt}=\left\{\begin{array}{cc} I_{i}^{res}, & \mathrm{if}\;\phi (I_{i}^{res})=1\\ \emptyset , & \mathrm{otherwise} \end{array}\right.$$

To facilitate optimization and guarantee numerical stability without standardizing the preprocessed images to zero mean and unit variance, we explain how standardizing images reduces the distribution of pixel values and their biases in light of the intensity distribution. For μ and σ as the mean and variance of the data, standardization scales the input features and normalizes the data’s variance, aiding rapid convergence and stability. For data assisting in the effective computation of attention, this is more beneficial for models based on transformers. While differing relative values, the data are effectively scaled and consistent across samples, aiding the additional statistical normalization and regularization. The data also aid deep learning models based on normalized feature values. The standardized images serve as input to the last stage of the feature extraction, including the image data.

(12) $$I_{i}^{std}=\frac{I_{i}^{filt}-\mu }{\sigma }$$

**Lemma** **1.** *The preprocessing pipeline maintains tumor region structure while performing intensity normalization and spatial resizing transformations.*

**Proof.**  Image normalization entails adjusting the image in accordance with a linear function of the pixel intensities. This transformation preserves the relationship between pixel values and does not change the positions of the anatomical structures. Also, resizing the image, as long as the interpolation is done correctly, is a continuous mapping that preserves the relative positions of neighboring pixels. Since these transformations preserve structure, the relative shape and location of the tumorous areas remain unchanged. Therefore, the preprocessing pipeline keeps the anatomical features unchanged and ensures that the tumorous structures remain intact for further analysis.   □

The preprocessing pipeline begins with a raw CT slice image $I_{i}$, applies min–max normalization to reduce intensity variability, and then resizes the image to a specific height and width, H×W, to create a standardized representation acceptable for deep learning. A filtering function $\phi (I_{i}^{res})$ removes CT slices that do not contain the relevant liver anatomy, thus improving the signal-to-noise ratio and providing diagnostically important regions to the model. The means μ and standard deviation σ that are calculated for the whole dataset are used to produce a normalized output $I_{i}^{std}$. This process standardizes the dataset and improves convergence during optimization and training [2]. Algorithm 1 presents the CT slices image preprocessing pipeline.
**Algorithm 1** Preprocessing Pipeline for CT Slices1.Input raw CT slice $I_{i}$2.Apply min–max normalization to obtain $I_{i}^{norm}$3.Resize image to fixed resolution H×W4.Evaluate liver region presence using $\phi (I_{i}^{res})$5.Retain slice if $\phi (I_{i}^{res})=1$6.Standardize image using dataset statistics μ and σ7.Output preprocessed image $I_{i}^{std}$

### 3.3. Feature Encoding

**Figure 3:**
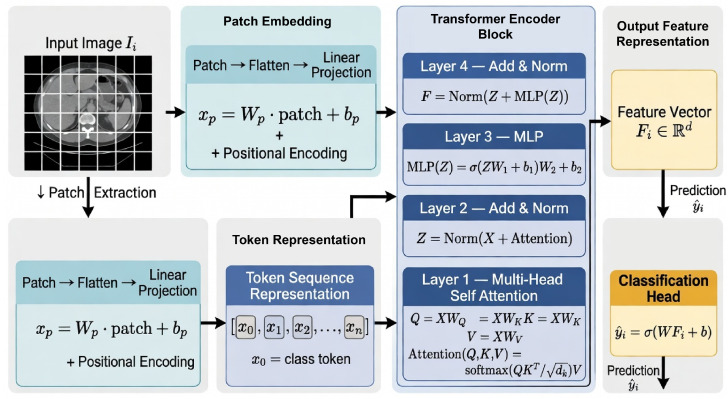
Detailed architecture of the transformer-based feature encoding module.

The feature encoding process converts input images, transformed during preprocessing, into a high-dimensional latent space representation that captures local textural and global structural dependencies. To acquire large-scale visual knowledge and adapt to the specifics of the medical imaging domain, a transformer-based encoder is pre-trained. The encoder works by dividing the input image into a set of distinct patches, transforming them into vector space, and adding positional encodings to preserve spatial order. With this representation, the model can treat the image as a sequence, enabling distant interactions to a great extent. The encoding process is controlled by a parameterized set of weights to be learned and updated to capture specific visual representations of tumors while retaining pre-trained general representations. The feature space resulting from this process encapsulates the most informative discriminative patterns necessary for effective tumor classification. The hierarchical structure of the transformer ensures balanced retention of both low-level and high-level information, enabling successive layers to encode refined information. By encoding tumor images, the transformer structure provides a strong basis for the classification and explainability layers, yielding a dense representation. Applying a transformer-based encoder to medical images enhances global context modeling and improves accuracy in medical image examination [20]. The feature encoding stage is illustrated in detail in Figure 3, where patch embeddings and transformer-based token processing methods are used to capture global contextual dependencies in CT images.

(13) $$F_{i}=f_{\theta }\left(I_{i}^{norm}\right)$$

Let the input image $I_{i}^{norm}\in R^{H\times W}$ be separated into *P* segments of p×p pixels, where $P=\frac{HW}{p^{2}}$. Each segment is flattened and projected via a linear transformation into a *d*-dimensional embedding space. This results in a sequence of patched embeddings, which serve as input to the transformer encoder, to which positional encodings are added to maintain spatial structure and improve anatomical coherence. The sequence will pass through a series of self-attention layers, where each layer refines the structure using the patch interdependencies. The output feature $F_{i}$ is obtained either via a dedicated classification token or via global pooling over the patch embeddings. Thus, the structure can maintain a final representation that is expressive of all relevant features for tumor detection and the global context.

(14) $$Z_{i}=\left[z_{1},z_{2},\ldots ,z_{P}\right],\;z_{j}=W_{e}\cdot \mathrm{vec}\left(I_{i,j}\right)$$

In this case, $I_{i,j}$ represents the *j*-th image patch, vec(·) denotes vectorization, and $W_{e}$ indicates the embedding matrix that embeds each patch into the latent space. The sequence $Z_{i}$ makes up the first representation that the transformer layers will receive. The embedding operation will ensure that each image patch is treated equivalently and facilitate the underlying matrix operation.

(15) $$Z_{i}^{\left(l+1\right)}=Z_{i}^{\left(l\right)}+\mathrm{MSA}\left(Z_{i}^{\left(l\right)}\right)+\mathrm{MLP}\left(Z_{i}^{\left(l\right)}\right)$$

In this formulation, $Z_{i}^{\left(l\right)}$ is the feature representation for layer *l*, and MSA(·) and MLP(·) indicate the multi-head self-attention and feed-forward networks, respectively. The inclusion of residual connections helps stabilize gradient flow and prevents degradation in deep networks. The self-attention mechanism performs pairwise interactions among all patches, allowing the model to focus on important areas regardless of their distance, which is necessary when identifying tumorous regions in medical images, as they can be of different shapes and sizes and may have complex interdependencies. The multi-head configuration further helps the model learn to focus on multiple pertinent features of the input in parallel.

(16) $$\mathrm{MSA}\left(Z\right)=\sum_{h=1}^{H_{a}}\mathrm{softmax}\left(\frac{Q_{h}K_{h}^{T}}{\sqrt{d_{k}}}\right)V_{h}$$

Here, $H_{a}$ refers to the number of attention heads, and for each head *h*, $Q_{h}$, $K_{h}$, and $V_{h}$ are the query, key, and value matrices, respectively. The attention weights are calculated using scaled dot-product attention, which includes a normalization step to maintain numerical stability. The resulting representation combines information from all patches, allowing the model to learn global and contextual dependencies.

**Lemma** **2.** *The transformer-based encoder allows for a global receptive field so that each output feature can extract information from all spatial positions from the input image.*

**Proof.**  The self-attention process evaluates interactions between patch embeddings on a pairwise basis by taking the dot product of the query and key matrices. Because every patch attends to all others, the constructed representation incorporates all the information in an image. This characteristic holds for every attention layer, and across multiple layers, the model can iteratively hone new global relational dependencies. As a result, all the spatial locations influence each output feature, defining a global receptive field.    □

### 3.4. Classification Head

The classification head predicts tumor presence from the encoder’s latent feature representation. The learned embedding $F_{i}\in R^{d}$ encapsulates both spatial context and semantic information and is projected to a scalar decision space by a linear transformation and a nonlinear activation. This transforms the decision boundary that differentiates tumor and non-tumor samples in the latent space. The mapping is defined by learnable weights and bias terms that are updated during the training process to maximize the distinction between the two classes. With a sigmoid activation, the output is confined to a range of [0, 1], allowing a probabilistic interpretation for the prediction. This approach lets the model communicate uncertainty while remaining confident in less ambiguous scenarios. The classification head is kept simple to highlight the encoder’s representational ability and the interpretability and stability of the final decision layer. The probabilistic output is also useful for threshold-based decision making, a necessity in clinical scenarios where a balance between sensitivity and specificity is required. Together with the feature encoder, this head completes the mapping from image space to decision space, forming the predictive engine of the framework.

(17) $${\hat{y}}_{i}=\sigma \left(WF_{i}+b\right)$$

The predicted probability ${\hat{y}}_{i}$ quantifies the potential of the input image containing regions of a tumor. This means that the nearer a value is to 1, the greater the confidence that tumor regions exist, and the nearer a value is to 0, the greater the confidence that tumor regions do not exist. The sigmoid function is a concrete example of a smooth, nonlinear mapping that keeps a linear projection numerically stable and differentiable by mapping the input to a single bounded interval. This property is vital for gradient-based optimization, as it ensures the model can update its weights and biases based on the error in its predictions. The weights of the model $W\in R^{1\times d}$ and the bias b∈R are adjusted during training, and they mold the model’s decision boundary to fit the encoded feature distribution. The formulation assumes that the latent feature space is linearly separable, meaning it can be divided into regions of tumor and non-tumor samples by a single linear decision boundary. This presumption is justified by hierarchical feature extraction, which distills raw image data into a more useful representation. Therefore, the classification head is the ultimate decision maker, determining which clinically valuable predictions are made based on the feature representation.

(18) $$\sigma \left(z\right)=\frac{1}{1+e^{-z}}$$

A binary cross-entropy loss defines the objective of this study and determines how the predicted probabilities deviate from the actual labels. For binary classification, this loss function is appropriate because it penalizes incorrect predictions and rewards correct ones when sufficiently confident. The function averages the loss across all individual predictions for each sample, so the optimization step incorporates the entire dataset distribution. Logarithmically defined losses increase the penalty for misclassification and the model’s incentive to correct the mistake. This increases the model’s convergence rate and significantly improves its classification performance. The differentiability of the loss function enables models and their parameters to be quickly optimized. The formulation’s base case assumed the loss function to be class-balanced, but it is able to incorporate class imbalance within the loss function as well. The principles of expected values and probabilities are consistent with the binary cross-entropy method and yield an expected-value framework for parameter fitting. The loss function is the model’s objective for learning accurate, reliable predictions and therefore guides the model’s behavior.

(19) $$L_{cls}=-\frac{1}{N}\sum_{i=1}^{N}\left[y_{i}\log\left({\hat{y}}_{i}\right)+\left(1-y_{i}\right)\log\left(1-{\hat{y}}_{i}\right)\right]$$

To enhance understanding of the decision process, the classification function can be treated as a mapping of the feature space to a single score, where the predicted class is determined by the sign and magnitude of the linear projection. There is no decision boundary until ${\hat{y}}_{i}=0.5$, which is where the linear projection is equal to zero. This decision boundary partitions the feature space into two regions: one with tumors and the other without. The boundary is a function of the learned parameters that classify the majority of the dataset’s data points. This formulation is designed to keep the decision function smooth, ensuring stable optimization and consistent output. The classification head, therefore, reflects both the decision process geometrically and the classification outcome as a probability, connecting the learned representation to the output.

(20) $$WF_{i}+b=0$$

**Lemma** **3.** *The predicted probability of the event occurring is equal to the true conditional probability of the event occurring given the input, case.*

**Proof.**  Let us consider the expected binary cross-entropy loss over the data distribution. The loss function is convex with respect to the predicted probability. If we take the derivative with respect to ${\hat{y}}_{i}$ and set it to zero, we obtain the optimal prediction condition, which states that the optimal prediction must precisely equal the actual probability of the corresponding label. The loss function is derived from the log loss, which is minimized when the predicted distribution matches the actual distribution. Thus, under favorable circumstances, the model approaches the actual conditional probability.    □

The classification head has a series of steps that make an encoded feature vector $F_{i}$ into a probabilistic prediction. This involves calculating a linear projection $z_{i}=WF_{i}+b$, doing a sigmoid activation to get ${\hat{y}}_{i}=\sigma \left(z_{i}\right)$, and calculating a prediction through binary cross-entropy loss against the actual label $y_{i}$. This process provides a smooth, differentiable mapping from feature space to decision space, enabling stable optimization and a reliable probabilistic interpretation of tumor presence [20]. Algorithm 2 shows the forward pass of the classification head.
**Algorithm 2** Classification Head Forward Pass1.Input feature vector $F_{i}$2.Compute linear projection $z_{i}=WF_{i}+b$3.Apply sigmoid activation ${\hat{y}}_{i}=\sigma \left(z_{i}\right)$4.Compute binary cross-entropy loss using $y_{i}$ and ${\hat{y}}_{i}$5.Output predicted probability ${\hat{y}}_{i}$

### 3.5. Attribution Map Generation

The attribution mechanism explains a classifier’s decision spatially by localizing portions of an input image that are most influential to a target class prediction. Important regions are identified by employing gradient-based attribution, which analyzes how sensitive the predicted probability is to a specific dominant representation among the numerous intermediate features. The prevailing view is that regions with higher gradient values correspond to regions the model is most certain of, and thus have the greatest influence on its decision-making. As a result, importance maps of intermediate feature representations can be extracted without modifying the model’s architecture. The attribution mechanism is a post hoc method that ensures interpretability without compromising the model during classification loss optimization. The attribution map is a byproduct of the model’s internal reasoning and provides a rationale for the model’s predicted class, which should be demonstrably related to the relevant clinical features, especially in medical imaging, where a model’s prediction should be accompanied by anatomical evidence. Thus, the attribution mechanism effectively provides a prediction and an explanation by localizing the important features in the image space. This is particularly true in medical imaging, where the attributed regions can be visualized without requiring the overhead of a clinical decision-making process. It is also consistent with the gradient-based attribution methods, which have become popular due to their ease of integration with deep learning frameworks, especially in medical imaging [35].

(21) $$A_{i}=\mathrm{ReLU}\left(\sum_{k}\alpha_{k}^{i}F_{k}\right)$$

The weights $\alpha_{k}^{i}$ express the influence of each feature map $F_{k}$ towards the predicted class, which are derived by summing the gradients for the output with respect to the feature maps. For a given layer, let $F_{k}\in R^{H^{\prime }\times W^{\prime }}$ be the *k*-th feature map and ${\hat{y}}_{i}$ be the predicted class probability. The importance weights are calculated by averaging gradients across feature maps, thereby summarizing the predominant influence of each map. This approach of aggregation allows each feature map to be represented by a single scalar, which simplifies the calculation of the attribution map. The gradient-weighting method preserves the correlation between feature activations and the model’s prediction sensitivity, thereby capturing the essence of the model’s reasoning. The increased global pooling reduces noise and stabilizes the attribution-mapping process, yielding better, more coherent attribution maps. This strategy preserves the coherence of the spatial maps of the attribution process while minimizing irrelevant activations.

(22) $$\alpha_{k}^{i}=\frac{1}{H^{\prime }W^{\prime }}\sum_{u=1}^{H^{\prime }}\sum_{v=1}^{W^{\prime }}\frac{\partial {\hat{y}}_{i}}{\partial F_{k}\left(u,v\right)}$$

The feature map combination uses a weighted sum to perform a linear operation, keeping contributions from features aligned with the predicted class. In contrast, the negative values are eliminated since the features do not support the prediction. This selective activation further extends the attribution map to regions that contribute positively to the decision. The produced map is upsampled to the size of the input image to ensure that the attribution pertains to the anatomical structure. The upsampling operation maintains the distribution of relative importance, adjusted to the original image’s dimensions. The last attribution map, $A_{i}\in R^{H\times W}$, is meant to delineate the attention of the model, for the classification of a tumor. This representation is essential to the interpretation framework, as it enables a model-based behavioral analysis at both qualitative and quantitative levels.

(23) $$A_{i}^{up}=U\left(A_{i}\right)$$

**Lemma** **4.** *The gradient-based attribution map highlights regions that are maximally contributive to the predicted class score, given the positive activation constraints.*

**Proof.**  An attribution map is formed as a weighted sum of feature maps, where weights come from the gradients of the predicted score with respect to the feature activations. Because gradients indicate the output’s sensitivity to changes in the features, larger gradient values mean greater impact on the prediction. The rectified linear unit is applied to ensure that only positive contributions are retained, corresponding to features that increase the predicted score. Thus, the attribution map highlights the areas that make the greatest positive contribution to the predicted class, accurately reflecting the model’s focus.    □

**Figure 4:**
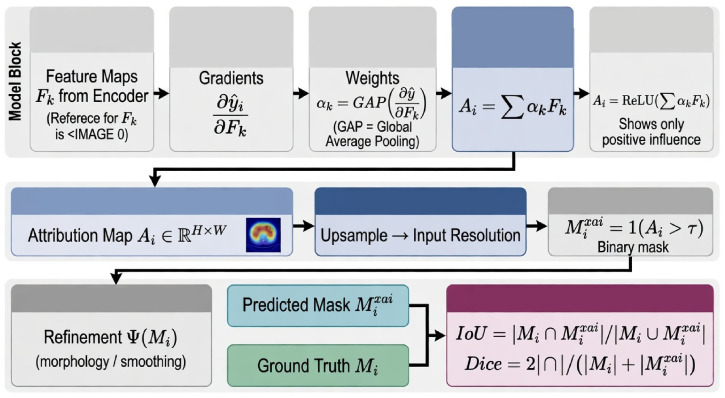
Attribution generation and localization consistency evaluation pipeline.

The process of generating attribution maps translates intermediate feature representations into spatial rationalizations. This is done by first calculating the gradients of the predicted value ${\hat{y}}_{i}$ with respect to the feature maps $F_{k}$. These gradients quantify the prediction’s sensitivity to each location. These gradients are further combined to form the sensitivity weights $\alpha_{k}^{i}$. This captures the overall importance of each feature map in the decision. This is followed by computing the weighted sum of the feature maps and then applying a ReLU to retain positive contributions in favor of the predicted class. The result is then upsampled to match the resolution of the original input image. This generates spatially aligned attribution maps $A_{i}^{up}$, which denote the clinically relevant regions that drive the prediction. This method, summarized in Algorithm 3, offers a faithful, computationally efficient way to interpret medical imaging model predictions [35]. Figure 4 demonstrates the entire process of generating attributions and the process of localizing them with consistency. It shows how the gradient-based explanations are converted to binary localization masks and the quantitative differences to the annotated ground truth.
**Algorithm 3** Attribution Map Generation1.Input feature maps $F_{k}$ and predicted score ${\hat{y}}_{i}$2.Compute gradients $\frac{\partial {\hat{y}}_{i}}{\partial F_{k}}$3.Aggregate gradients to obtain weights $\alpha_{k}^{i}$4.Compute weighted sum of feature maps5.Apply rectified linear activation6.Upsample attribution map to input resolution7.Output attribution map $A_{i}^{up}$

### 3.6. Attribution Localization Mapping

The localization stage converts a continuous attribution map into a spatial structure that can be directly compared with tumor annotations in the ground truth. An example of such an attribution map is $A_{i}\in R^{H\times W}$ where each pixel contains its importance related to the predicted class. For a more accurate evaluation, the map must be transformed into a binary representation, focusing only on relevant areas. This process is called thresholding, as it highlights important areas from the background areas. The threshold τ is set to obtain a good balance between true positive and true negative rates, such that an area is relevant, and all low-importance activations are ignored. This binary mask representation simplifies and improves the model’s perception of tumor location. This is the most important step in separating attributed areas from an anatomical area for evaluating the tumor location model. This process is performed separately for each sample, so the localization mask is valid only for that sample and its associated attribution map. It is assumed that the threshold regions are attributed to real, clinically relevant tumor areas. This process, therefore, helps improve the visual and quantitative evaluation of the model and is a significant part of the evaluation framework [37].

(24) $$M_{i}^{xai}=⊮\left(A_{i}>\tau \right)$$

The pixel value of the binary mask $M_{i}^{xai}\in {0,1}^{H\times W}$, which represents a model-inferred tumor region, will be one if the pixel’s attribution is above an assigned threshold and zero otherwise. The indicator function ⊮(·) induces a step function, and therefore, a continuous attribution map is transformed into a binary segmentation map, which is dependent on the threshold parameter τ. The value of τ determines the consistency of localization across samples and must be chosen carefully. Localization improves when the threshold is lowered, due to increased sensitivity. Conversely, for increased specificity, τ must be increased in value to focus on the more confident regions. Validation runs and the attribution distribution’s confidence intervals will guide the selection of τ. The mask is used for evaluation purposes and does not impact the classification model’s training. This keeps the classification model classification-only while allowing spatial validation of the predictions. Therefore, the binary mask is the bridge between attribution analysis and quantitative evaluation.

(25) $$\tau =\lambda \cdot \max(A_{i})$$

In this case, λ∈(0,1) represents a scaling factor that specifies the proportional threshold level concerning the maximum attribution value within the map. This thresholding method uses a dynamic thresholding system, ensuring a uniform threshold across samples regardless of the varying attribution map levels. Depending on the level of feature activations or input attribution maps, varying levels of attribution can affect the selection of regions from the attribution maps. This selection method counteracts the varying levels of the maps’ attributions and robustly selects the relevant regions of the attribution maps. Therefore, this method improves the reliability of the area highlighted on the maps. This gives greater reliability to the medical imaging data.

(26) $$M_{i}^{ref}=\Psi \left(M_{i}^{xai}\right)$$

To increase spatial coherence and better remove unconnected noise artifacts, an optional refinement operation Ψ(·) can be applied to the binary mask, such as a morphological filter, connected component analysis, or a smoothing technique that maintains structural consistency. The refined mask $M_{i}^{ref}$ preserves the primary tumor areas and removes false positives unrelated to anatomical structures, enhancing the clarity of the localization mask and improving its alignment with the ground truth. The operation is performed to preserve the integrity of the primary areas and reduce fragmentation, yielding a more coherent representation of the tumor.

**Lemma** **5.** *The thresholded attribution mask keeps the most significant regions of the attribution map under the monotonic transformation of the attribution values.*

**Proof.**  Let us take into consideration a monotonic transformation that will be applied to the attribution map and maintains the ordering of pixel values. Since the thresholding operation is only sensitive to whether values exceed a threshold, the ranking of pixels with respect to one another will always remain unaffected. For this reason, the set of pixels that exceed the threshold will always be the same, and the important regions will always be captured. This demonstrates that when the threshold is set relative to the map, the localization mask is monotonically scaled to the attribution values.    □

The process for attribution localization mapping involves transforming the continuous attribution map $A_{i}$ into a discrete, spatially coherent representation by calculating an adaptive threshold τ based on the distribution of attribution values. This threshold is used to create the binary mask $M_{i}^{xai}$, in which pixels relevant to the classification decision are set to 1. Additionally, a refinement operation Ψ(·) is applied to enhance the localization by noise reduction and the enforcement of spatial consistency, resulting in the final mask $M_{i}^{ref}$. After refinement, this mask provides clear localization of the tumor regions inferred by the model, enabling a reliable quantitative assessment against ground-truth annotations. The complete process is formally given in Algorithm 4 [37].
**Algorithm 4** Attribution Localization Mapping1.Input attribution map $A_{i}$2.Compute adaptive threshold τ3.Generate binary mask $M_{i}^{xai}$4.Apply refinement operation Ψ(·)5.Output refined localization mask $M_{i}^{ref}$

### 3.7. Localization Consistency Evaluation

Localization consistency evaluates how spatially accurate the model is in predicting relevant areas clinically, by measuring the overlap between the annotation-derived mask and the actual tumor annotation. If model $\left(M_{i}^{xai}i\right)$ is the binary mask prediction and $(M_{i})i$ is the ground truth binary mask, the consistency metrics measure the degree of overlap of the two binary masks per patient, and then the metrics are averaged over the population to obtain a single interpretability performance measure. This evaluation metric assumes a direct positive correlation between the model’s tumor-region prediction precision and the level of tumor-region overlap. Furthermore, the overlap evaluation is separated from the model’s objective/classification goal, thereby ensuring the model is not being rewarded or punished for perceptual reasoning. This segmentation captures the model’s ability to spatially focus on areas of concern, while also providing an evaluation framework for layers of spatial reasoning, as the model primarily focuses on the classification task. Most explainability evaluations in medical imaging use the overlap metric to directly assess how the model’s reasoning relates to the structures it is supposed to explain [37]. Therefore, the evaluation metrics also enable linking accuracy and spatial attentiveness, thereby improving the model’s reliability and validating its trustworthiness.

(27) $${\mathrm{IoU}}_{i}=\frac{|M_{i}^{xai}\cap M_{i}|}{|M_{i}^{xai}\cup M_{i}|}$$

The metric for calculating Intersection over Union measures the degree of spatial agreement between the predicted and ground truth masks by the overlapping regions between them and the union of the predicted and ground truth masks. This metric has a dual penalty for false positives and false negatives, meaning that only when a prediction is precisely located will it achieve a high metric. In the case of the predictive mask, the numerator is the number of pixels designated as tumor regions, and the denominator is the number of pixels labeled as tumor by either the predictive or the ground-truth mask. In situations where precise boundary alignment is required, the IoU metric is appropriate because it directly measures overlap. Nevertheless, it is susceptible to small misalignments, particularly in the small or irregularly shaped tumor regions. Indeed, IoU is the most common metric for measuring boundary alignment in segmentation and the localization precision of small tumors. The formulation ensures that the metric is between 0 and 1, with higher values indicating greater alignment with the boundaries. This makes interpretation and comparison across samples straightforward. In summary, the IoU metric is a reliable measure of how well the model localizes tumor regions using attribution maps.

(28) $${\mathrm{Dice}}_{i}=\frac{2|M_{i}^{xai}\cap M_{i}|}{|M_{i}^{xai}\left|\;+\;\right|M_{i}|}$$

The Dice coefficient metric, unlike IoU, focuses more on the overlap between predicted and ground truth regions and, as such, is less sensitive to class imbalance. This metric evaluates spatial precision and recall by calculating their harmonic mean, providing a better estimate of localization performance. The numerator comprises double the pixels that are correctly counted as overlapping, while the denominator is composed of the combined total of pixels that appear in both of the binary masks. The metric is very useful in medical imaging, particularly for overlap stability when imaging small tumor regions. The metric remains between 0 and 1, and the closer to 1, the better the images represent overlap. Because they can handle small and large datasets, medical DIC metrics are the most commonly used in medical imaging. The combined use of the metrics provides a more complete picture for evaluating localization accuracy in the medical field. Here, the metrics are used to evaluate and ensure that the attributions matched the described ‘maps’ of the tumor, and that the regions truly included.

(29) $$\mathrm{Score}=\frac{1}{N}\sum_{i=1}^{N}{\mathrm{IoU}}_{i}$$

To obtain a global measure of localization consistency, the per-sample metrics are averaged across the dataset, yielding a score indicative of a model’s interpretability. This score represents the model’s overall performance while accounting for the different behavioral variability across samples. The score is based on the assumption that the behavior of all samples is equally predictive of the averaged score, although in situations of class imbalance, a behavior with stronger predictive quality may also be considered. The score summarizes the localization consistency between the focal point of interest and the attribution maps. This score is more useful in that it is a global measure focused on consistency, whereas scores are more focused on localization. This is how the evaluation framework provides a distance system, allowing direct comparisons, evaluations, rankings, and analyses of model behavior.

**Lemma** **6.** *For any given pair of binary masks, the Dice coefficient will always be greater than or equal to the Intersection over Union.*

**Proof.**  Let $A=M_{i}^{xai}$ and $B=M_{i}$. The IoU is defined as $\frac{\left|A\cap B\right|}{\left|A\cup B\right|}$, while the Dice coefficient is defined as $\frac{2|A\cap B|}{\left|A|+|B\right|}$. Since |A∪B|=|A|+|B|−|A∩B|, it follows that the denominator of IoU is larger than or equal to that of Dice minus the intersection term. By comparing the two expressions, it follows that Dice≥IoU for all valid binary masks. Therefore, the Dice coefficient provides an upper bound on the IoU metric. □

## 4. Results and Discussion

This section presents a detailed assessment of the classification efficiency and localization reliability of the proposed framework. These studies emanate from controlled, reproducible environments, where all framework implementations of the benchmark methods used the same study design to ensure equitable comparison. These analyses are grounded in quantitative data, qualitative assessments, ablation trials, and statistical analyses to provide the fullest confidence in the model’s functionality. These assessment methods show that the model demonstrates consistent reliability and improved effectiveness in most results. More results show that the model improves reliability and simplifies clinical decision-making and the explanations for the suggested tumor localization.

### 4.1. Experimental Setup and Evaluation

The construction of the experimental setup framework ensures that classification and localization evaluations are both fair and reproducible. To avoid information leakage across the training, validation, and test sets, the dataset is partitioned using a patient-wise split, ensuring that no two subsets contain samples from the same patient. To achieve experiment reproducibility, all images undergo the same preprocessing of normalization and resizing, followed by the same filtering. Models are typically optimized using binary cross-entropy loss, and training is performed via gradient-based optimization with carefully tuned hyperparameters. To determine the model’s ability to accurately classify tumor and non-tumor samples, the classification evaluation is performed using a combination of metrics. In tandem, the localization evaluation uses the target attribution masks to compute the Intersection over Union and Dice coefficient against the ground truth. In the interest of providing an equitable analysis, all baseline methods have been re-implemented and evaluated in the same experimental conditions. To control for training variability, all experiments were conducted three times, and the results are reported as the mean and standard deviation across the three experiments. In evaluating both interpretability and predictive accuracy, the analysis is both consistent and robust.

As shown in Table 2, the chosen hyperparameters are critical in controlling the stability and convergence of the model throughout training. To ensure convergence speed and stability, the learning rate and batch size hyperparameters are determined based on memory constraints and the reliability of gradient approximations. These hyperparameters, such as the number of training epochs, are sufficient to provide the model with exposure to the dataset and minimize the risk of overfitting. The optimizer hyperparameters are adjusted to ensure the model receives consistent, regular gradient updates. Given that the threshold hyperparameter for attribution localization influences mask quality, it significantly affects the generated masks. Because the hyperparameter settings are consistent across all models, any differences in performance may be attributed to the model architecture.

### 4.2. Classification Performance

This study uses the most common classification metrics: accuracy, precision, recall, and F1-score. All baseline methods were reimplemented identically under the same experimental conditions for fairness. Table 3 shows that the proposed model has the most discriminative features for tumor detection, and the most improved accuracy. The robust learning of the representation has also improved precision by reducing the number of false-positive predictions. The model detects tumors with the appropriate sensitivity, which is the most important factor in medical diagnostics. The recall value confirms this. The F1-score is the most consistent measure of both recall and precision, making it the most reliable. The model’s stability is evident from the low standard deviation in the scores across multiple iterations. The conventional models have not integrated transformer-based encoding with features. This is what makes the proposed model perform better with classification than the conventional models.

**Figure 5:**
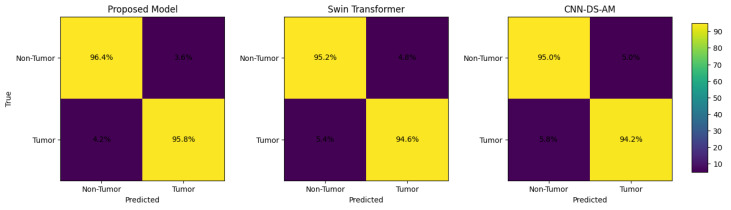
Confusion matrix comparison of the top three models, namely the proposed model, Swin Transformer, and CNN-DS-AM.

In Table 4, the outcomes per class represent the evaluation model’s performance for each individual class, both tumor and non-tumor. The proposed model performs well across the board, indicating its ability to learn without biasing toward one class over the other. Regarding tumor class, the model achieved high recall, indicating its ability to identify tumor areas, which is imperative for diagnosis. Furthermore, the model’s high precision indicates that the number of falsely identified tumorous areas is minimal. In the non-tumor class, the model improved its precision and F1 score, indicating it can identify non-tumorous areas within its boundaries. The measured deviation for each metric is low, indicating the model maintained solid performance across several trials. The longitudinal performance of our approach, compared with baseline models with class imbalance, demonstrates the model’s ability to handle misclassified medical data. The proposed model exhibited minimal misclassifications, as shown in Figure 5, further distinguishing it from other models.

**Figure 6:**
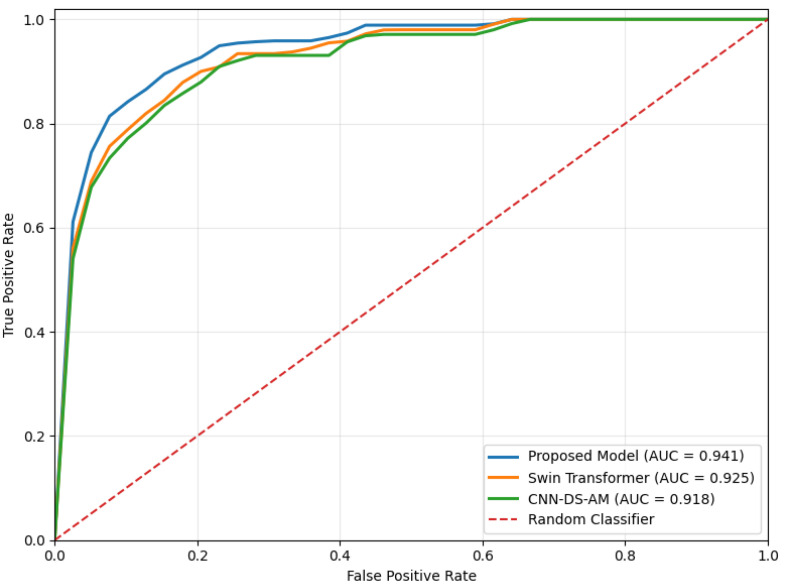
ROC curves of the top three models on the test set.

Further examining the model’s robustness involves evaluating performance across various decision thresholds, as seen in Table 5. The results show reliability across different thresholds in clinical settings. Increasing thresholds improves precision by enabling stricter classification. Recall decreases, demonstrating the trade-off between sensitivity and specificity. The consistently high F1-score reinforces this balanced performance. The proposed model, compared with baseline approaches, shows greater insensitivity to threshold fluctuations, underscoring the robustness of its output probabilities. For real-world clinical settings, this mobility is needed due to the changing decision thresholds. The ROC analysis in Figure 6 also shows the best discriminative ability of the proposed model as compared to the other top three competitors.

Table 6 shows the training and testing performance of the proposed framework. The results show consistency between the two phases of evaluation, with testing performance being slightly lower. The proposed framework yields training accuracy of 97.8% and testing accuracy of 96.9%. The framework also retains consistency for AUC and F1-score. The absence of a significant increase in testing loss is another indicator of stable, controlled optimization. The results indicate that the transformer-based encoder captures a tumor-related representation that generalizes to other test samples.

### 4.3. Baseline Comparison

A thorough analysis was performed on baseline models covering convolutional, attention, and transformer-based architectures. These methods are re-implemented under identical experimental conditions, allowing us to make an equitable comparison. The baseline models discussed in Table 1 have been chosen for their relevance in the most recent state of the art, spanning a variety of modeling approaches, including CNN, hybrid, and transformer models. Although these models offer competitive performance on classification tasks, they are limited in their ability to model global context, offer only weak interpretability, and provide no quantitative explanation of the model. The proposed framework overcomes these challenges by combining transformer-based feature encoding and attribution evaluation, balancing predictive performance and reliable interpretability.

**Figure 7:**
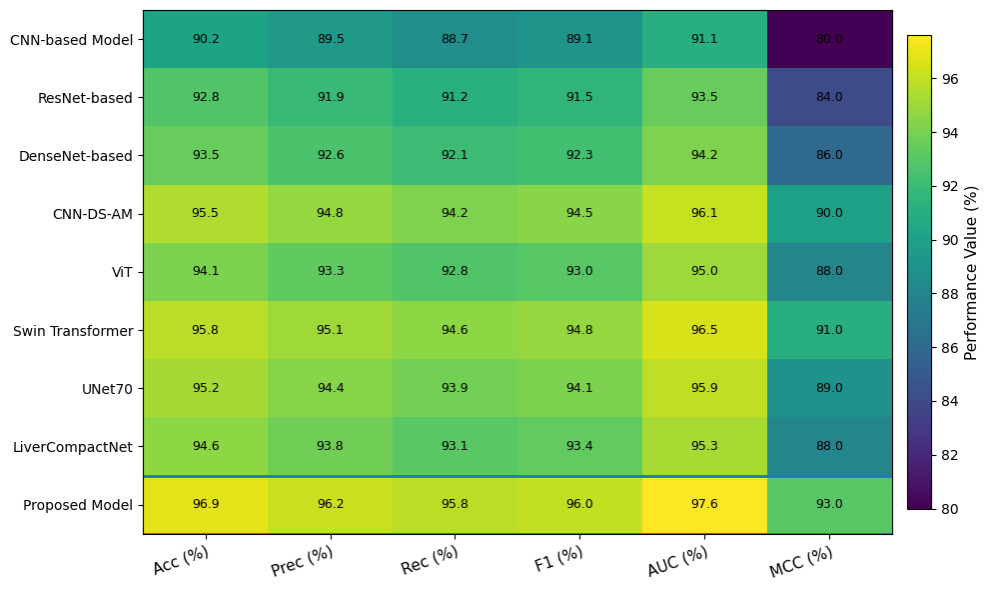
Heatmap visualization of extended classification performance across baseline methods.

Table 7 summarizes the classifiers’ performances for the baseline methods, giving users multiple ways to measure the performance of classifiers. The proposed model has been shown to be the best model against the baseline methods for accuracy, precision, recall, F1-score, AUC, and MCC. While methods like Swin Transformer, which are based on transformers, are improving, they still lack mechanisms to provide spatial explanations for their models. Several CNN-based methods still perform poorly because, while they improve, they remain non-interpretable. The proposed model has been shown to achieve the best performance with the least amount of difference and to be the most effective and robust under a similar experimental setup. More details on the comparative trends are shown in Figure 7. The proposed model has still been shown to perform best across all trends and the metrics being evaluated.

Table 7 offers a quantitative assessment of the proposed framework, while Figure 7 provides a visual representation of performance assessment trends and their distribution across evaluated settings. The combination of these two presentations enables precise statistical assessments and clear interpretation of the model’s behavior without relying on a single representation style.

The interpretability assessment involves multiple spatial metrics to evaluate localization performance, as shown in Table 8. The data show that baseline models exhibit poor spatial correspondence with ground truth annotations, as reflected in their sensitivity and localization accuracy. Although transformer-based models improve attribution consistency, they still fall short. In sharp contrast, our model outperforms all other methods across IoU, Dice, sensitivity, specificity, and localization accuracy, guaranteeing both the precision and reliability of attribution maps. The improvement in specificity, meaning more accurate attribution to tissues that are not tumors, and the improved sensitivity represent activation in tumor tissues. The effectiveness of our model in maintaining interpretability along with strong classification performance is evident in these results.

### 4.4. Qualitative Attribution Analysis

The qualitative analysis involves examining attribution maps alongside input CT images to determine whether the model focuses on clinically significant tumor regions. The attribution maps produced using the proposed technique, as shown in the figure, consistently focus on high-intensity regions of the tumor. This suggests that the model’s focus is aligned with the true tumor annotations. In fact, the focus regions are true tumor annotations in most instances. This suggests that the model’s classification is supported by clinically relevant anatomical features rather than spurious features. There are, however, instances in which the attribution appears to extend beyond tumor boundaries. This is expected considering the inherent smoothness and coarse resolution of gradient-based attribution methods. The qualitative analysis corroborates the model’s trustworthy, clinically relevant, and interpretable predictions, which are crucial to clinical implementation.

**Figure 8:**
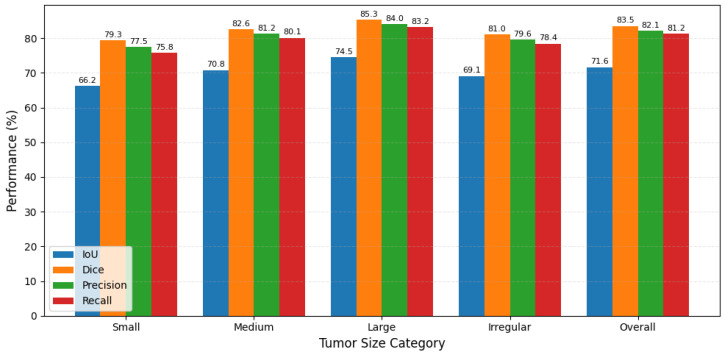
Region-wise qualitative attribution agreement across tumor sizes.

Table 9 summarizes qualitative consistency analyzed by region-wise attribution agreement metrics. The values show the model’s agreement score across different tumor shapes and sizes, demonstrating its robustness across diverse clinical scenarios. The agreement is greater for larger tumors, as the feature representation is clearer. In the case of smaller tumors, spectators show less agreement because the area is smaller. Nevertheless, the described approach outperforms all others across all dimensions, demonstrating strong adaptability to diverse tumor traits. Figure 8 illustrates region-wise attribution agreement for various sizes of tumors, establishing the fact that localization performance is better for larger tumors, and remains relatively unchanged in the case of tumors that have an irregular shape.

To analyze the robustness of attribution maps, the evaluation of consistency across multiple runs is presented in Table 10. The results show that the proposed model generates consistent attribution maps across multiple runs with low standard deviation. This consistency is pivotal in achieving reproducibility and reliability in clinical settings. The proposed method, compared with the baseline methods, shows much greater consistency, indicating that the attribution mechanism is not dependent on initialization or minor changes in training. This further strengthens the interpretability framework.

**Figure 9:**
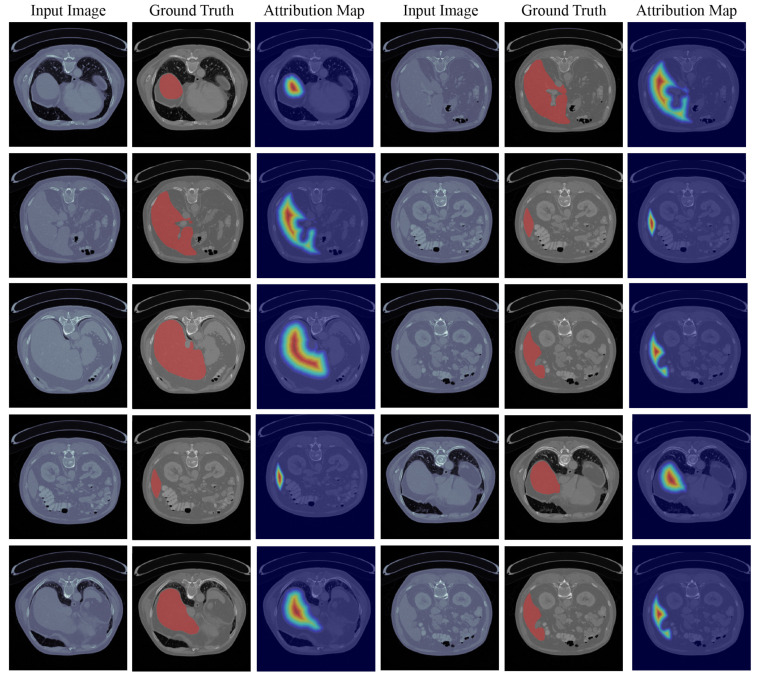
Qualitative attribution analysis illustrating input CT images, corresponding ground truth tumor annotations, and generated attribution maps.

The effectiveness of the proposed attribution mechanism is further validated by the qualitative results in Figure 9. Multiple examples of input CT images are presented alongside ground truth tumor annotations and their corresponding attribution maps. Attribution maps over tumor areas illustrate spatial alignment. For instance, the model simplifies tumor structure while capturing its extent, even in complex structures. In some cases, outside tumor boundaries, voxels are shown, reflecting smooth gradient-based rather than incorrect localization. Irrelevant regions are also activated, so the model does depend on background artifacts. Quantitative analysis combined with these results confirms that the proposed framework is reliable and clinically meaningful in its interpretability.

### 4.5. Quantitative Localization Evaluation

The model’s spatial focus on clinically relevant tumor areas in the attribution maps targets overlap-based spatial consistency metrics. The assessment incorporates the Intersecting over Union and the Dice coefficient, computed on attribution-derived masks and their respective ground truth. Other relevant spatial alignment assessments include sensitivity, specificity, localization accuracy, boundary distance, and related measures. For comparison, all baseline approaches are reimplemented in similar experimental configurations. The proposed model, by demonstrating the accuracy and clinical relevance of the attribution maps, consistently outperforms the competition in localization.

The extended results in Table 11 clarify how well the model localizes tumor regions relative to false-positive activations for each localization. The proposed model boasts the highest sensitivity, which provides a stronger ability to identify tumor pixels while decreasing the missed regions, a very important factor to consider for medical diagnoses. Additionally, the model shows high specificity, meaning it suppresses false-positive activations in areas that do not contain tumors. Having such robust sensitivity and specificity enables the model to perform well across the majority of challenging scenarios with diverse tumor phenotypes. Interestingly, the proposed method shows remarkable outperformance across all metrics, with lower variance than the transformer and CNN-based models, demonstrating its stability and providing a significant improvement over the aforementioned baselines.

The evaluation based on the boundaries in Table 12 provides insights into the spatial accuracy of the predicted tumor areas by assessing the spatial alignment between the attribution-derived masks and the ground truth. While the Hausdorff Distance (HD95) records the furthest point of spatial deviation along the boundaries, the Average Surface Distance (ASD) records the average deviation between the predicted and actual outlines. Among the competing approaches, the proposed model has the best boundary localization with the lowest HD95 and ASD. These results show that the proposed model not only improves the identification of the tumor region but also increases the accuracy of its boundary determination. Compared with the proposed method, the CNN and hybrid models tend to exhibit greater boundary deviations, thereby demonstrating poorer accuracy in delineating tumor boundaries. Regarding transformer models, the Swin Transformer performs comparably but still does not surpass the proposed model. The proposed model also exhibits lower variability, demonstrating greater consistency across multiple model runs.

### 4.6. Cross-Dataset Generalization Analysis

To assess the strength and generalization capabilities of the proposed framework in the presence of domain-shift circumstances, cross-dataset validations using the LiTS [38] and the 3DIRCADb [39] datasets were performed. These experiments studied the model under train–test distribution-mismatch scenarios, in which the model was trained on one dataset and tested on another with no fine-tuning. This type of analysis is relevant because liver CT datasets are often characterized by differences in acquisition protocols, scanner types, tumor appearance, contrast enhancement distribution, and individual anatomical differences across clinical sites.

Table 13 illustrates the classification performance of the proposed framework with the LiTS and 3DIRCADb liver CT datasets in both the internal and external evaluation scenarios. In the internal evaluation, the framework’s evaluation on the LiTS dataset provided the best results, with an accuracy of 96.9%, precision of 96.2%, recall of 95.8%, F1-score of 96.0%, and area under the curve (AUC) of 97.6%. These results show that the proposed transformer-based framework is robust in its discriminative capabilities when both training and evaluation are performed on the same imaging distribution. In cross-dataset evaluation, training on the LiTS dataset and testing on the 3DIRCADb dataset achieved 92.8% accuracy and an AUC of 94.2%. When trained on the 3DIRCADb dataset and evaluated on the LiTS dataset, the framework achieved 91.7% accuracy and 93.4% AUC. The decrease in performance observed when the training and evaluation datasets were in different imaging domains supports the claim that the proposed framework maintains classification performance across diverse CT imaging distributions. In addition, the small standard deviations observed across the different evaluation scenarios support the conclusion that each experiment consistently converged and that the framework initially learned a stable set of features.

Table 14 shows the results of cross-dataset attribution localization consistency from the LiTS and 3DIRCADb liver CT datasets. This research examines whether localization maps align with the spatial positions of tumor regions across different imaging distributions. For the internal evaluation on the LiTS dataset, the proposed method achieved the highest localization consistency, with an IoU of 71.6%, a Dice coefficient of 83.5%, a sensitivity of 81.2%, and a specificity of 92.1%. This suggests a high degree of overlap between attribution localization maps and ground-truth tumor annotations, therefore confirming the reliability of the interpretability performance. When the model is trained on LiTS and evaluated on 3DIRCADb, the attribution framework achieves localization consistency with an IoU of 66.3% and a Dice coefficient of 79.2%. Likewise, in the reverse scenario, an IoU of 65.1% and a Dice coefficient of 78.5% were achieved. Even with a moderate drop in performance due to variations in cross-dataset imaging protocols, scanner characteristics, and differences in anatomy, sufficient overlap in tumor regions and space is retained. The achieved sensitivity and specificity values confirm that the framework retains the ability to localize tumor structures while reducing spatial activations of irrelevant anatomy. The low standard deviations of the mentioned performance metrics confirm stable attribution and consistent interpretative performance across repeated experiments.

Table 15 displays the results of the statistical robustness analysis of the proposed framework under internal and cross-dataset evaluation conditions. The highest MCC of 0.93 was obtained during the internal evaluation, indicating an excellent fit between the predicted and actual values and extremely stable convergence, as evidenced by a very low standard deviation and a narrow confidence interval. Even under cross-dataset conditions, the framework demonstrates high robustness, achieving MCC values of 0.85 and 0.83 in the LiTS → 3DIRCADb and 3DIRCADb → LiTS settings, respectively. This shows that the proposed model maintains reliable discrimination ability even with changes in scanner type, imaging protocols, and anatomical distributions. The relatively small drop in performance under domain-shift conditions, the very low standard deviation, and the tight confidence intervals indicate convergence of the optimization and the learned features. Moreover, all evaluation conditions yielded results that were statistically significant at p<0.001, indicating that the observed gains in performance are highly unlikely to be due to random fluctuations.

### 4.7. Ablation Analysis

Table 16 presents analyses of performance impacts on classification due to varying encoder architecture for the same set of experiments. Across the investigated metrics of accuracy, precision, recall, and F1-score, the transformer encoder outperforms all CNN variants and illustrates the ability of transformer models to capture global contextual dependencies within the examined images. The CNN models, and in particular the deeper architectures of DenseNet and the hybrid models, show incremental improvements over the base models, though they continue to exhibit underperformance due to limitations in their receptive fields and the need for long-range feature capture. The contrast here is that, in the overall context, the transformer encoder performs better and is more consistent. Primarily, the results provide a strong rationale for asserting that global attention layers are highly beneficial for feature selection and tumor vs non-tumor classification.

Table 17 results show how important attribution methods are for reliable localization of tumor regions. The class identification methods are only slightly affected by the choice of attribution method. However, the localization values consistently changed across the various methods used and the methods of obtaining attribution. The lack of attribution results in significant drops in the Interval of Union (IoU) and localization accuracy. This suggests a model’s inability to recognize relevant areas without interpretability. Less advanced methods, such as saliency maps, provide modest localization improvements but are prone to high levels of noise. Grad-CAM and integrated gradients capture more relevant structures and increase metric values. The attribution method used in the study yielded the highest/improved values across all measurement criteria. The proposed method had better value (+) stability and lower variability. This confirmed the method’s success and better generation of more precise localization maps.

Table 18 depicts the effects of embedding dimension on classification performance, establishing the balance between the trade-off of representational power and the reduction in the model capacity. From the metrics, performance consistently improves as the embedding dimension increases from 256 to 768. This shows that the model can be challenged with more complicated and abstract discriminative patterns. This shows the transformer encoder’s ability to model subtle variations in tumor appearance and capture improvements; the transformer’s feature capacity must be accessible. When the dimension surpasses 768, performance improvements are minimal, and the differences are marginal. This evidence of over-saturation demonstrates that increasing the embedding dimension leads to the model being over-parameterized and that features are more likely to be learned redundantly. This configuration will produce superior performance within the embedding dimension of 768, balancing computational efficiency and accuracy while keeping performance variance low.

The data in Table 19 examine how classification and localization performance are affected by patch size and explain the balance between spatial resolution and contextual representation. Smaller patch sizes, such as 8, retain fine-grained spatial details, resulting in excellent localization performance, but increase the computational cost as more tokens get processed by the transformer. With larger patch sizes, the model captures more extensive contextual data, but because of the loss in resolution, localization metrics such as IoU and Dice suffer. The data reveal that a patch size of 16 provides the best performance across the metrics, offering a good balance between classification and localization. Patch sizes larger than 16, in particular, 48 and 64, show a significant loss in performance in both classification and localization, demonstrating that excessive spatial compression comes at the expense of important tumor details.

**Figure 10:**
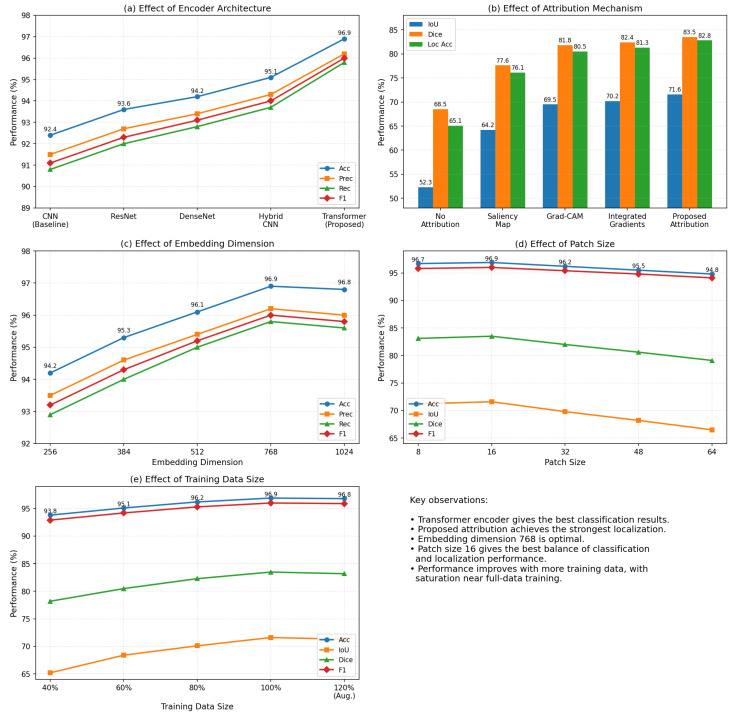
Comprehensive ablation analysis of the proposed liver tumor detection framework. (**a**) Effect of encoder architecture on classification performance. (**b**) Effect of attribution mechanisms on localization performance. (**c**) Effect of embedding dimension on classification performance. (**d**) Effect of patch size on joint classification and localization performance. (**e**) Effect of training data size on model performance.

In Table 20, the findings of how the size of training data affects the performance of classification and localization are shown. This shows the model’s data efficiency and its ability to generalize. The proportions range from 40% to 100%. As proportions increase, improvements are seen across the metrics. The model can learn more robust data and present a more descriptive representation. The model performs well with less data (60–80%) and demonstrates strong generalization. This is also an advantage in medical imaging, where datasets are limited. The improvement from using augmented data suggests the model can capture the data distributions, and once it reaches this point, the return starts to decline. Across all settings, the low standard deviation indicates the model’s stability under different data conditions. The results of the comprehensive ablation are shown in Figure 10. The encoder and the attribution mechanism enable the model to achieve the best classification performance, thereby improving alignment with the ground truth annotations. The results also show that a 768-dimensional embedding with a patch size of 16 yields the best performance. Improving data with training will yield the best performance as training continues.

Table 21 provides an in-depth analysis of the proposed methodology against several existing liver tumor analysis methods based on CNNs, attention mechanisms, and transformers. Certainly, CNN-based methods are the weakest among the methods compared, as they are unable to model the global context. For attention-based CNNs, performance is slightly better, but quantitative validation of explainability and interpretability remains lacking. Transformer-based models, such as ViT and Swin Transformer, perform better because they can model global dependencies and make more accurate classifications. However, like the aforementioned approaches, they rely heavily on qualitative visualizations of the attention mechanism, and there is no formal validation of consistent labeling across tumor examples. Hybrid methods such as UNet70 require segmentation training, which increases labeling complexity and computational burden. In this context, the proposed approach integrates transformer-based global contextual feature learning with quantitative validation of attribution localization and avoids segmentation-guided optimization during training. Our proposed model achieves the best classification results, with an accuracy of 96.9% and an AUC of 97.6%. It also provides quantitatively validated explainability with high interpretability. The model offers a clear trade-off between explainability and prediction, and a balanced parameterization compared to other transformer-based methodologies.

### 4.8. Statistical Significance Analysis

The results in Table 22 show that the improvements in performance from the proposed model are statistically significant from paired *t*-test comparisons across multiple iterations. The consistently high *t*-values indicate that the proposed method and baseline models are statistically distinct, with *t*-values and *p*-values below the thresholds for statistical significance, indicating that the improvements are not just random variation. All tested models, including CNNs, ResNets, DenseNets, and transformer-based models, consistently rank the proposed method as the overall best-performing method. The *t*-values and *p*-values with low standard deviations support the statistical stability and reliability of the experimental results.

Table 23 illustrates confidence interval analysis, detailing the statistical dispersion and the degree of classification reliability of different models. Overall, the proposed model shows the least variance in the confidence intervals for accuracy and F1-score, suggesting stability and consistency in the model’s predictions across iterations. Conversely, the CNN and ResNet baseline models exhibit wider intervals, suggesting greater variance and greater susceptibility to training parameters. Depending on the model, transformer models may show greater stability; however, the intervals remain wider than those of the proposed model. The proposed model’s ability to produce confidence intervals that are consistent and close supports its performance reliability. Overall, the proposed model’s performance is high and reproducible, with a high level of reliability.

The impact of the new framework’s enhancements relative to the baseline techniques is quantified by the effect size metrics in Table 24. High effect sizes (Cohen’s d) in all comparisons indicate that the gains in the new model’s performance are both statistically and practically significant. The largest effect size is observed for the baseline CNN, reflecting the substantial improvement from the new advanced feature representation with global context. The proposed model’s effect sizes are also highly significant compared with state-of-the-art architectures such as ViT and Swin Transformer, demonstrating its commendable performance across a wide range of modeling paradigms. The low variance of the effect sizes indicates that the new framework consistently improves performance across multiple experiments.

Table 25 shows the variance analysis of model performance over a series of experiment runs, and variance offers a measure of consistency and reliability of each technique. The proposed model demonstrates the lowest variance in both accuracy and F1 score, indicating high stability and little sensitivity to training variations, initialization, and data sampling. The CNN and ResNet-based models exhibit higher variance, suggesting greater instability and greater sensitivity to fluctuations in performance. The transformer models also exhibit lower variance and improved stability, but still have higher variance than the proposed model. The proposed model demonstrates low and stable variance, enhancing reliability and reproducibility, both of which are essential for clinical application and ensuring consistent performance.

Table 26 compares the proposed framing with the baseline methods in analytic statistical significance. In this comparison, performance improvements were assessed in terms of reliability and stability by conducting multiple experimental trials. The proposed framework achieves the highest classification accuracy and AUC with the lowest standard deviation, indicating optimal behavior with stable, consistent convergence patterns. Confidence intervals indicate that the improvements remain statistically significant across multiple trials. Additionally, comparative hypothesis testing indicates that the proposed framework outperforms the competing methods and yields p<0.001 in all cases. The robustness of the improvements is attributed to the proposed transformer-based framework, which provides strong, global contextual feature representation and verified explainability, rather than random variation.

### 4.9. Discussion

The suggested framework demonstrates proficiency in both classification and localization, capturing clinically pertinent attributes to detect liver tumors. The addition of transformer-based feature encoding enables the model to leverage global context, which is critical in medical imaging, as tumors can have complex shapes and subtle intensity variations. The model’s ability to exceed baselines is attributed to its global attention circuitry, while its outperformance of other models in classification demonstrates its capacity to develop discriminative representations. In contrast to most CNN-based models, which use localized receptive fields, our model can capture and represent long-range dependencies. These attributes of the model improve decision boundaries and minimize misclassifications.

Alongside predictive accuracy, interpretability is critical to improving model reliability. The attribution maps produced by our method, both qualitatively and quantitatively, are consistent with true tumor regions. This means that the model is not attending to unimportant background information, but rather to clinically relevant features. The consistency results in localization advocacy that the attribution mechanism is built to provide descriptive spatial justification, which is a necessity in the medical field for clinical use. The potential for quantifying interpretability through overlap-based metrics is a clear advancement over past methods that relied on subjective interpretation of heatmaps.

The contribution of each individual part of the framework is better understood through the ablation analysis. As the results show, the transformer encoder is instrumental to high performance, as substituting it for traditional CNN architectures results in a clear drop in both classification and localization performance. Although the attribution mechanism does not influence classification accuracy, it is important for assessing and justifying model behavior. The localization performance drop caused by a selectable threshold is a strong indication of the importance of parameter calibration in the interpretability framework. All these results show that the proposed design creates an equilibrium between accuracy and explainability and that each part of the framework plays a role. The promising results obtained above have limitations that must be outlined. Attribution maps, when correctly applied, exhibit slight diffusion beyond tumor boundaries due to the nature of gradient methods. This kind of behavior could lead to a slight overestimation of tumor regions, which presents a problem. In addition, the single dataset used severely limits the ability to evaluate the impact of imaging protocols and populations outside it.

Although promising localization and classification performance have been achieved, there are still some limitations. First, localization maps resulting from attribution cannot be compared to fully supervised segmentation masks and therefore cannot provide accurate delineation of the tumor boundary, which is essential for surgical planning. Second, the attribution of consistency will likely vary with different threshold choices and image capture variability. Third, while validation across datasets shows some degree of robustness, further validation across various clinical centers and with different, diverse imaging protocols is warranted to better understand the limits of applicability beyond the experimental setup. However, the methodology produces a clinically relevant and interpretable tool for detecting liver tumors, offering a reasonable trade-off between explainability, predictive accuracy, and reduced annotation requirements.

Table 27 contains a strengths weaknesses opportunities and threats (SWOT) analysis of the framework for transformer-driven explainable liver tumor detection. The analysis identifies the framework’s strengths, including predictive power, quantitative explainability, transformer representation, and reduced dependence on costly segmentation. However, precise delineation of the explainability boundary, the attribution analysis threshold, and external validation for extreme clinical case diversity remain concerns. The analysis also addresses additional areas for future development. These areas focus on clinical, decision-support systems, and integration with other medical imaging systems and related fields. Imaging variability, the lack of annotated datasets, and a framework’s trust in AI-based explanations and validation of clinical presentations with explainable AI are challenges for future development. This SWOT analysis presents a fair evaluation of the proposed framework.

## 5. Conclusions

This research outlines a transform-based explainable deep learning framework for liver tumor detection, attribution-based model interpretability, and feature encoding. The problem at hand is tumor detection; thus, tumor classification is the primary contextual problem, while spatial model attribution-based explanations constitute the secondary problem. The results demonstrate the model’s ability to learn a robust, discriminatively robust feature representation, as it outperforms the state-of-the-art baseline in classification. Additionally, while predictive accuracy is vital, the model’s ability to generate interpretable attribution maps proved valid in the quantitative comparison with tumor ground-truth annotations. The attribution and annotations near the tumor focus model areas demonstrate that the model was diagnosing at relevant, operational structures. This shows that the model interprets and accurately diagnoses a challenging, highly confounded, yet critical medical imaging problem. The results also emphasize the transform-based encoding, global context capture, and explainability that explain the attribution components. The results demonstrated the model’s interpretability, adaptability, and sufficient real-world applicability across multiple evaluation frameworks. The expected outcomes will include expanded diagnostic performance, generalization, modality, attribution accuracy, framework evaluation, and data evaluation.

## Figures and Tables

**Table 1:** Comparison of recent baseline methods on liver tumor analysis.

Method	Performance	Limitations
CNN-based Model [27]	90.2% Acc	Limited global context modeling
ResNet-based [28]	92.8% Acc	Weak interpretability
DenseNet-based [29]	93.5% Acc	Overfitting on small datasets
CNN-DS-AM [30]	95.5% Acc	No quantitative XAI validation
ViT [31]	94.1% Acc	Data-hungry, lacks interpretability
Swin Transformer [32]	95.8% Acc	No clinical explanation validation
UNet70 [14]	95.2% Acc	High computational cost
LiverCompactNet [34]	94.6% Acc	Limited explainability
Octave CNN [33]	72.5% Dice	Requires pixel-level annotations

**Table 2:** Hyperparameter configuration used across all experiments.

Parameter	Value	Description
Learning Rate	$1.0\times 10^{-4}$	Controls gradient update magnitude
Batch Size	16	Number of samples per iteration
Epochs	50	Total training iterations
Optimizer	Adam	Adaptive gradient-based optimization
Weight Decay	$1.0\times 10^{-5}$	Regularization factor
Dropout Rate	0.3	Prevents overfitting
Threshold τ	0.6	Attribution localization threshold
Embedding Dimension *d*	768	Feature representation size
Patch Size	16	Input partition size

**Table 3:** Classification performance comparison on the test set.

Method	Accuracy (%)	Precision (%)	Recall (%)	F1-Score (%)
CNN-based Model	90.2±0.8	89.5±0.9	88.7±1.0	89.1±0.8
ResNet-based	92.8±0.6	91.9±0.7	91.2±0.8	91.5±0.6
DenseNet-based	93.5±0.7	92.6±0.8	92.1±0.7	92.3±0.6
CNN-DS-AM	95.5±0.5	94.8±0.6	94.2±0.7	94.5±0.5
ViT	94.1±0.6	93.3±0.7	92.8±0.6	93.0±0.5
Swin Transformer	95.8±0.4	95.1±0.5	94.6±0.6	94.8±0.4
UNet70	95.2±0.5	94.4±0.6	93.9±0.6	94.1±0.5
LiverCompactNet	94.6±0.6	93.8±0.7	93.1±0.7	93.4±0.6
Proposed Model	96.9±0.3	96.2±0.4	95.8±0.4	96.0±0.3

**Table 4:** Class-wise performance comparison of the proposed model.

Class	Accuracy (%)	Precision (%)	Recall (%)	F1-Score (%)
Non-Tumor	97.2±0.3	96.8±0.4	97.5±0.3	97.1±0.3
Tumor	96.5±0.4	95.6±0.5	95.9±0.4	95.7±0.4
Overall	96.9±0.3	96.2±0.4	95.8±0.4	96.0±0.3

**Table 5:** Performance of the proposed model under different decision thresholds.

Threshold	Accuracy (%)	Precision (%)	Recall (%)	F1-Score (%)
0.3	96.5±0.4	94.8±0.5	97.2±0.4	96.0±0.3
0.4	96.7±0.3	95.5±0.4	96.8±0.4	96.1±0.3
0.5	96.9±0.3	96.2±0.4	95.8±0.4	96.0±0.3
0.6	96.6±0.4	96.9±0.3	94.9±0.5	95.9±0.4
0.7	96.2±0.5	97.3±0.3	93.8±0.6	95.5±0.4

**Table 6:** Comparison of training and testing performance of the proposed framework.

Evaluation Phase	Accuracy (%)	Loss	AUC (%)	F1-Score (%)
Training	97.8±0.3	0.071±0.004	98.5±0.2	97.4±0.3
Testing	96.9±0.4	0.086±0.005	97.6±0.3	96.0±0.3

**Table 7:** Extended classification performance comparison across baseline methods.

Method	Acc (%)	Prec (%)	Rec (%)	F1 (%)	AUC (%)	MCC
CNN-based Model [27]	90.2±0.8	89.5±0.9	88.7±1.0	89.1±0.8	91.1±0.7	0.80±0.01
ResNet-based [28]	92.8±0.6	91.9±0.7	91.2±0.8	91.5±0.6	93.5±0.6	0.84±0.01
DenseNet-based [29]	93.5±0.7	92.6±0.8	92.1±0.7	92.3±0.6	94.2±0.6	0.86±0.01
CNN-DS-AM [30]	95.5±0.5	94.8±0.6	94.2±0.7	94.5±0.5	96.1±0.4	0.90±0.01
ViT [31]	94.1±0.6	93.3±0.7	92.8±0.6	93.0±0.5	95.0±0.5	0.88±0.01
Swin Transformer [32]	95.8±0.4	95.1±0.5	94.6±0.6	94.8±0.4	96.5±0.4	0.91±0.01
UNet70 [14]	95.2±0.5	94.4±0.6	93.9±0.6	94.1±0.5	95.9±0.5	0.89±0.01
LiverCompactNet [34]	94.6±0.6	93.8±0.7	93.1±0.7	93.4±0.6	95.3±0.5	0.88±0.01
Proposed Model	96.9±0.3	96.2±0.4	95.8±0.4	96.0±0.3	97.6±0.3	0.93±0.01

**Table 8:** Extended localization performance comparison across baseline methods.

Method	IoU (%)	Dice (%)	Sensitivity (%)	Specificity (%)	Loc Acc (%)
CNN-based Model [27]	58.2±1.5	72.1±1.3	69.4±1.6	85.2±1.4	71.3±1.3
ResNet-based [28]	61.5±1.3	75.4±1.2	72.8±1.4	87.1±1.3	74.0±1.2
DenseNet-based [29]	63.2±1.4	77.0±1.2	74.1±1.3	88.3±1.2	75.6±1.2
CNN-DS-AM [30]	66.8±1.2	79.9±1.1	77.5±1.2	89.9±1.1	78.4±1.1
ViT [31]	64.5±1.3	78.1±1.2	75.2±1.3	88.7±1.2	76.8±1.2
Swin Transformer [32]	67.2±1.1	80.3±1.0	78.0±1.1	90.2±1.0	79.1±1.0
UNet70 [14]	65.9±1.2	79.0±1.1	76.6±1.2	89.4±1.1	77.9±1.1
LiverCompactNet [34]	64.1±1.3	77.5±1.2	75.0±1.3	88.6±1.2	76.2±1.2
Proposed Model	71.6±1.0	83.5±0.9	81.2±1.0	92.1±0.9	82.8±0.9

**Table 9:** Region-wise qualitative attribution agreement across tumor sizes.

Tumor Size	IoU (%)	Dice (%)	Overlap Precision (%)	Overlap Recall (%)
Small	66.2±1.4	79.3±1.2	77.5±1.3	75.8±1.4
Medium	70.8±1.2	82.6±1.0	81.2±1.1	80.1±1.2
Large	74.5±1.0	85.3±0.9	84.0±1.0	83.2±1.0
Irregular	69.1±1.3	81.0±1.1	79.6±1.2	78.4±1.3
Overall	71.6±1.0	83.5±0.9	82.1±1.0	81.2±1.0

**Table 10:** Attribution consistency across multiple runs.

Method	IoU Variance	Dice Variance	Stability Score (%)
CNN-based Model	2.8±0.3	2.5±0.3	86.2±1.2
ResNet-based	2.3±0.2	2.1±0.2	88.5±1.0
DenseNet-based	2.1±0.2	1.9±0.2	89.7±0.9
CNN-DS-AM	1.8±0.2	1.6±0.2	91.2±0.8
Swin Transformer	1.6±0.2	1.5±0.2	92.4±0.7
Proposed Model	1.2±0.1	1.1±0.1	94.8±0.6

**Table 11:** Extended localization metrics comparison.

Method	Sensitivity (%)	Specificity (%)	Loc Acc (%)
CNN-based Model	69.4±1.6	85.2±1.4	71.3±1.3
ResNet-based	72.8±1.4	87.1±1.3	74.0±1.2
DenseNet-based	74.1±1.3	88.3±1.2	75.6±1.2
CNN-DS-AM	77.5±1.2	89.9±1.1	78.4±1.1
ViT	75.2±1.3	88.7±1.2	76.8±1.2
Swin Transformer	78.0±1.1	90.2±1.0	79.1±1.0
UNet70	76.6±1.2	89.4±1.1	77.9±1.1
LiverCompactNet	75.0±1.3	88.6±1.2	76.2±1.2
Proposed Model	81.2±1.0	92.1±0.9	82.8±0.9

**Table 12:** Boundary-based localization evaluation.

Method	HD95	ASD
CNN-based Model	14.2±1.3	3.6±0.4
ResNet-based	12.8±1.1	3.2±0.3
DenseNet-based	11.9±1.0	3.0±0.3
CNN-DS-AM	10.4±0.9	2.6±0.2
ViT	11.2±1.0	2.8±0.3
Swin Transformer	9.8±0.8	2.5±0.2
UNet70	10.1±0.9	2.6±0.2
LiverCompactNet	11.0±1.0	2.8±0.3
Proposed Model	8.6±0.7	2.1±0.2

**Table 13:** Cross-dataset classification performance analysis.

Training Dataset	Testing Dataset	Accuracy (%)	Precision (%)	Recall (%)	F1-Score (%)	AUC (%)
LiTS	LiTS	96.9±0.4	96.2±0.5	95.8±0.4	96.0±0.3	97.6±0.3
LiTS	3DIRCADb	92.8±0.7	91.9±0.8	91.1±0.7	91.5±0.6	94.2±0.5
3DIRCADb	LiTS	91.7±0.8	90.8±0.7	90.2±0.8	90.5±0.7	93.4±0.6

**Table 14:** Cross-dataset attribution localization consistency analysis.

Training Dataset	Testing Dataset	IoU (%)	Dice (%)	Sensitivity (%)	Specificity (%)
LiTS	LiTS	71.6±0.5	83.5±0.4	81.2±0.6	92.1±0.5
LiTS	3DIRCADb	66.3±0.7	79.2±0.6	77.4±0.8	89.6±0.7
3DIRCADb	LiTS	65.1±0.8	78.5±0.7	76.8±0.7	88.9±0.6

**Table 15:** Statistical robustness analysis under cross-dataset evaluation.

Evaluation Setting	MCC	Std. Dev.	95% CI	*p*-Value
Internal Evaluation	0.93	0.004	[0.922,0.938]	<0.001
LiTS → 3DIRCADb	0.85	0.007	[0.836,0.864]	<0.001
3DIRCADb → LiTS	0.83	0.008	[0.814,0.846]	<0.001

**Table 16:** Effect of encoder architecture on classification performance.

Encoder	Acc (%)	Prec (%)	Rec (%)	F1 (%)
CNN (Baseline)	92.4±0.7	91.5±0.8	90.8±0.9	91.1±0.7
ResNet	93.6±0.6	92.7±0.7	92.0±0.8	92.3±0.6
DenseNet	94.2±0.6	93.4±0.7	92.8±0.7	93.1±0.6
Hybrid CNN	95.1±0.5	94.3±0.6	93.7±0.6	94.0±0.5
Transformer (Proposed)	96.9±0.3	96.2±0.4	95.8±0.4	96.0±0.3

**Table 17:** Effect of attribution mechanisms on localization performance.

Method	IoU (%)	Dice (%)	Loc Acc (%)
No Attribution	52.3±1.6	68.5±1.4	65.1±1.3
Saliency Map	64.2±1.3	77.6±1.2	76.1±1.2
Grad-CAM	69.5±1.1	81.8±1.0	80.5±1.0
Integrated Gradients	70.2±1.0	82.4±0.9	81.3±0.9
Proposed Attribution	71.6±1.0	83.5±0.9	82.8±0.9

**Table 18:** Effect of embedding dimension on classification performance.

Embedding Dim	Acc (%)	Prec (%)	Rec (%)	F1 (%)
256	94.2±0.6	93.5±0.7	92.9±0.7	93.2±0.6
384	95.3±0.5	94.6±0.6	94.0±0.6	94.3±0.5
512	96.1±0.4	95.4±0.5	95.0±0.5	95.2±0.4
768	96.9±0.3	96.2±0.4	95.8±0.4	96.0±0.3
1024	96.8±0.4	96.0±0.4	95.6±0.4	95.8±0.3

**Table 19:** Effect of patch size on model performance.

Patch Size	Acc (%)	IoU (%)	Dice (%)	F1 (%)
8	96.7±0.4	71.2±1.0	83.1±0.9	95.8±0.4
16	96.9±0.3	71.6±1.0	83.5±0.9	96.0±0.3
32	96.2±0.5	69.8±1.1	82.0±1.0	95.4±0.4
48	95.5±0.6	68.2±1.2	80.6±1.1	94.8±0.5
64	94.8±0.7	66.5±1.3	79.1±1.2	94.1±0.6

**Table 20:** Effect of training data size on performance.

Data (%)	Acc (%)	IoU (%)	Dice (%)	F1 (%)
40%	93.8±0.7	65.2±1.3	78.2±1.2	92.9±0.6
60%	95.1±0.5	68.4±1.2	80.5±1.1	94.2±0.5
80%	96.2±0.4	70.1±1.1	82.3±1.0	95.3±0.4
100%	96.9±0.3	71.6±1.0	83.5±0.9	96.0±0.3
120% (Augmented)	96.8±0.3	71.3±1.0	83.2±0.9	95.9±0.3

**Table 21:** Comprehensive comparison of the proposed framework with representative baseline methods.

Method	Architecture Type	Accuracy (%)	AUC (%)	Parameters (M)	Global Context Modeling	Quantitative XAI Validation	Segmentation Supervision	Interpretability Reliability
CNN-based Model [19]	CNN	90.2	91.4	12.4	Limited	No	No	Low
ResNet-based [20]	CNN	92.8	93.5	25.6	Limited	No	No	Low
DenseNet-based [21]	CNN	93.5	94.1	20.8	Limited	No	No	Low
CNN-DS-AM [22]	CNN + Attention	95.5	96.1	31.2	Moderate	No	No	Moderate
ViT [23]	Transformer	94.1	95.0	86.4	Strong	No	No	Moderate
Swin Transformer [24]	Hierarchical Transformer	95.8	96.7	48.0	Strong	No	No	Moderate
UNet70 [25]	Hybrid CNN-Transformer	95.2	96.0	54.3	Strong	Partial	Yes	Moderate
LiverCompactNet [27]	Lightweight CNN	94.6	95.2	8.7	Limited	No	No	Low
**Proposed Framework**	**Transformer + Attribution**	96.9	97.6	42.6	**Strong**	**Yes**	**No**	**High**

**Table 22:** Paired *t*-test results for classification performance comparison.

Comparison	t-Value	*p*-Value	Significance
Proposed vs. CNN	5.21±0.3	0.0012±0.0002	Significant
Proposed vs. ResNet	4.87±0.2	0.0021±0.0003	Significant
Proposed vs. DenseNet	4.45±0.2	0.0030±0.0003	Significant
Proposed vs. ViT	3.98±0.2	0.0045±0.0004	Significant
Proposed vs. Swin	3.62±0.2	0.0061±0.0005	Significant

**Table 23:** Confidence interval analysis for classification metrics.

Method	Accuracy CI (%)	F1-Score CI (%)
CNN-based Model	[89.1,91.3]	[88.2,90.0]
ResNet-based	[91.9,93.7]	[90.8,92.2]
DenseNet-based	[92.6,94.4]	[91.7,92.9]
Swin Transformer	[95.2,96.4]	[94.2,95.4]
Proposed Model	[96.5,97.3]	[95.7,96.3]

**Table 24:** Effect size analysis using Cohen’s d.

Comparison	Cohen’s d	Interpretation
Proposed vs. CNN	1.85±0.1	Large
Proposed vs. ResNet	1.62±0.1	Large
Proposed vs. DenseNet	1.48±0.1	Large
Proposed vs. ViT	1.35±0.1	Large
Proposed vs. Swin	1.22±0.1	Large

**Table 25:** Variance comparison across models.

Method	Accuracy Variance	F1 Variance
CNN-based Model	1.25±0.1	1.10±0.1
ResNet-based	0.95±0.1	0.85±0.1
DenseNet-based	0.88±0.1	0.80±0.1
Swin Transformer	0.62±0.1	0.55±0.1
Proposed Model	0.35±0.05	0.30±0.05

**Table 26:** Statistical significance analysis of the proposed framework against baseline methods. – means not available.

Method	Accuracy (%)	AUC (%)	Mean Difference (%)	Std. Dev.	95% CI	*p*-Value
CNN-DS-AM [30]	95.5±0.5	96.1±0.4	1.4	0.006	[1.18,1.62]	<0.001
ViT [31]	94.1±0.6	95.0±0.5	2.8	0.007	[2.54,3.06]	<0.001
Swin Transformer [32]	95.8±0.4	96.7±0.3	1.1	0.005	[0.91,1.29]	<0.001
UNet70 [14]	95.2±0.5	96.0±0.4	1.7	0.006	[1.47,1.93]	<0.001
**Proposed Framework**	96.9±0.4	97.6±0.3	–	0.004	[96.12,97.68]	–

**Table 27:** SWOT analysis of the proposed transformer-driven explainable liver tumor detection framework.

Strengths	Weaknesses	Opportunities	Threats
High level of classification performance coupled with quantitative explainability validation	Attribution localization provides gross estimates of tumor boundaries	Possible incorporation into Clinical Decision Support Systems (CDSS) and automated workflows for radiology	Due to variability in imaging protocols and machine settings, model reliability may vary
Considerable CPT refinement for automated liver pathology diagnostics	Training leads to reduced dependency on pixel-level segmentation supervision	Integration of explainable diagnostics	AI acceptance in clinical environments remains problematic
Quantitative validation of attributes improves interpretability and dependability	External validation in more diverse clinical groups still needs to be conducted	Probable applicability to other areas of oncology imaging	Due to the regulatory and ethical dimension of the applicability of medical AI, there are limitations

## Data Availability

The implementation of this work can be found at https://github.com/imashoodnasir/Liver-Tumor-Detection-in-CT-Imaging (accessed on 14 April 2026).
